# Transcriptional and post-transcriptional regulation of the jasmonate signalling pathway in response to abiotic and harvesting stress in *Hevea brasiliensis*

**DOI:** 10.1186/s12870-014-0341-0

**Published:** 2014-12-02

**Authors:** Julien Pirrello, Julie Leclercq, Florence Dessailly, Maryannick Rio, Piyanuch Piyatrakul, Kuswanhadi Kuswanhadi, Chaorong Tang, Pascal Montoro

**Affiliations:** CIRAD, UMR AGAP, F-34398 Montpellier, France; Rubber Research Institute, Chatuchak, Bangkok 10900 Thailand; Sembawa Research Centre, Indonesian Rubber Research Institute, P.O 1127, Palembang, 30001 Indonesia; Rubber Research Institute, Chinese Academy of Tropical Agricultural Sciences, Danzhou, 571737 Hainan China

**Keywords:** Latex, Tapping panel dryness, Jasmonic acid, Alternative splicing, Rubber, Transcriptional regulation

## Abstract

**Background:**

Latex harvesting in *Hevea brasiliensis* amounts to strong abiotic stress that can cause a halt in production in the most susceptible clones. Although the role of jasmonic acid has been suggested in laticifer differentiation, its role in latex production and in the response to harvesting stress has received very little attention. Only a few key genes acting in the COI-JAZ-MYC module have been isolated and studied at transcriptional level.

**Results:**

Use of a reference transcriptome obtained on rubber clone PB 260 covering a large number of tissues under different environmental conditions enabled us to identify 24 contigs implicated in the jasmonate signalling pathway in the rubber tree. An analysis of their expression profile by qPCR, combined with hierarchical clustering, suggested that the jasmonate signalling pathway is highly activated in laticifer cells and, more particularly, in the response to harvesting stress. By comparison with their genomic sequences, the existence of regulation by alternative splicing was discovered for JAZ transcripts in response to harvesting stress. Lastly, positive transcriptional regulation of the *HbJAZ_1405* gene by MYC was demonstrated.

**Conclusion:**

This study led to the identification of all actors of jasmonate signalling pathway and revealed a specific gene expression pattern in latex cells. In-depth analysis of this regulation showed alternative splicing that has been previously shown in *Arabidopsis*. Interestingly, genotypic variation was observed in *Hevea* clones with contrasting latex metabolism. This result suggests an involvement of jasmonate signalling pathway in latex production. The data suggest that specific variability of the JA pathway may have some major consequences for resistance to stress. The data support the hypothesis that a better understanding of transcriptional regulations of jasmonate pathway during harvesting stress, along with the use of genotypic diversity in response to such stress, can be used to improve resistance to stress and rubber production in *Hevea*.

**Electronic supplementary material:**

The online version of this article (doi:10.1186/s12870-014-0341-0) contains supplementary material, which is available to authorized users.

## Background

Jasmonic acid (JA) is a key hormone in the development of plant responses to abiotic stress, such as wounding [1]. This plant hormone also plays a key role in the biosynthesis of secondary metabolites [2]. It is particularly involved in the response to oxidative stress since it activates the ascorbate-glutathion cycle for the reduction of these major antioxidants [3,4].

The jasmonate biosynthesis and signalling pathways have been very widely studied and described in *Arabidopsis* using the analysis of mutants [5]. The *jar1* mutant is deficient in certain responses to JA, including ascorbate production to prevent oxidative stress [6]. The JAR1 enzyme catalyses the conjugation of jasmonate to isoleucine (JA-Ile) [7]. JA-Ile is the bioactive form of JA [8]. It was demonstrated recently that COI1 is the JA receptor and can interact directly with JA-Ile [9]. For its part, the *coi1* mutant also shows insensitivity to JA, thereby causing male sterility, resistance to the inhibition of root development by JA, and a defect in the expression of genes regulated by JA [10]. COI1 is an F-box protein [11] forming, with some other partners, a complex of the E3 ubiquitin ligase type, SCF^COI1^ [12,13]. This type of complex is involved in the ubiquitination of target proteins, leading to their subsequent degradation by the 26S proteasome [14]. JASMONATE ZIM DOMAIN (JAZ) proteins are recognized by the SCF^COI1^ complex. JAZ degradation enables the release of transcription factors such as MYC2,3 and 4 [15]. The latter bind to *cis*-acting elements of the G-box type [16] present in the promoters of JA response genes and they initiate their transcription [17-19]. In addition to the MYC transcription factors, JAZ are able to interact with other transcription factors of the MYB, EIN3, EIL, ERF, GAI, RGA and RGL1 types, suggesting that they play a role in the interaction of the JA signalling pathway with those of other hormones [20,21]. In *Arabidopsis*, JAZ proteins comprise 12 members which are characterized by the presence in the C-terminal position of the highly conserved domains, Jas and ZIM (TIFY). The Jas domain enables interaction with COI1 and with transcription factors, while the TIFY domains involved in dimerization and in the interaction with NINJA [21-23]. At the moment, the most likely model concerning the repression of genes induced by JA is an interaction of JAZ with TOPLESS (TPL). This interaction may necessitate the presence of the NINJA protein as an adapter, unless the JAZ protein possesses an EAR domain (ERF-associated amphiphilic repressor) to which TPL binds [1]. The transcriptional regulation of *JAZ* can occur via MYC2 [16], but other components might be involved. Indeed, in the *myc2* mutant, most *JAZ* were expressed following infection with *Pseudomonas syringae* [24]. The sub-unit of the mediator complex, MEDIATOR25 (MED25), was recently identified as an integrative node of JA-dependent gene expression [25]. At post-transcriptional level, most *JAZ* genes can be regulated by alternative splicing. In *Arabidopsis*, it has been found that intron/exon organization in the region of the Jas domain is conserved in the majority of *JAZ* genes [26]. In *Arabidopsis,* the spliced form of JAZ10 has a Jas domain that is partially (JAZ10.3) or totally truncated (JAZ10.4) [23]. Ectopic expression of JAZ10.3 and JAZ10.4 affords dominant insensitivity to JA as a consequence of the stabilization of the JAZ protein [19,23]. The physiological role of isoforms with a truncated Jas domain in attenuating hormonal response has been suggested by various studies [19,27]. Many studies have shown the importance of alternative splicing in plant development, but also in the response to stress (for review [28]), though that link has never been demonstrated in the case of post-transcriptional regulation of *JAZ* genes.

*Hevea brasiliensis* is the only commercial source of natural rubber (NR). NR is synthesized in the rubber particles of latex cells. Those cells differentiate from the cambium then anastomose to form an articulated network: laticifers. Latex is harvested by tapping the tender bark (phloem tissues). After latex flow, rubber particles agglomerate and clog the tapping zone. Latex is regenerated within a few days. In order to stimulate latex production 2.5% ethephon (an ethylene releaser) is applied to the tapping panel. Tapping and stimulation frequency is usually adapted to the metabolic activity of *Hevea* clones. In extreme cases of environmental or man-made stress due to rubber tree tapping, an oxidative burst occurs in the laticifers. That oxidative stress leads to peroxidation of lipids in the membrane of lutoids (poly dispersed lysosomal vacuome), which contain agglutinins such as heveins. The release of these factors leads to the *in situ* coagulation of rubber particles: this is the physiological syndrome known as Tapping Panel Dryness (TPD) [29]. TPD causes substantial NR production losses (10-40%) and economic models predict that NR production will not be sufficient by 2020. The genetic variability existing within the different cultivated rubber clones shows that the intrinsically most productive and fast growing clones are also the most susceptible to abiotic stress and TPD [30]. Some rubber clones with a slow laticifer metabolism are more tolerant of TPD. To date, few selected clones have the latter characteristics [31]. For instance, clone PB260 is a clone with an active metabolism that is highly susceptible to TPD, while INC53 and RRIM600 are clones with a slower metabolism and are more resistant to TPD. The physical wounding caused by tapping, the osmotic shock within the laticifers due to latex flow, and metabolic activation linked to latex regeneration between two tappings amount to harvesting stress which causes diverse responses, including the production of hormones such as JA. In fact, wounded tissues produce systemin, which induces JA production [32]. Interestingly, jasmonate and wounding are also involved in laticifer differentiation [33]. It was recently shown that jasmonate acts as a signal molecule in rubber biosynthesis [34]. Although the jasmonate signalling pathway has been studied in the rubber tree in connection with rubber biosynthesis and laticifer differentiation, little is known about its role in the response to harvesting stress. To date, only the ethylene biosynthesis and signalling pathways have been largely studied in this connection, and notably the transcriptional regulation of *ERFs* (Ethylene Response Factor) [35-39]. In *Hevea,* one or two members of the multigene families encoding COI [40], JAZ [41] and MYC [42] have been identified. Studies on the expression profile of those genes suggest the importance of JA in latex production. Indeed, *HbCOI1* is strongly expressed in laticifers [40], the transcripts of *HbJAZ1* accumulate in response to tapping and wounding [41], and *HbMYC1* and *HbMYC2* are more abundant in latex. *HbMYC1* is induced by regular tapping and wounding, while the expression of *HbMYC2* is not altered by those stimuli [42].

In this study, the availability of *Hevea* transcriptomic and genomic resources made it possible to identify all the different genes acting in the jasmonate signalling pathway and characterize their implication during development, and in response to abiotic stress. Among the transcriptomes sequenced on different tissues (latex, bark, leaves and stem tips) [43-48], a reference transcriptome covering a large number of tissues and environmental conditions has been published for rubber clone PB 260 [35]. An analysis of the structure of *JAZs* genes based on the genomic sequences of rubber clone CATAS 7-33-97 (BIG-CATAS Project, unpublished data), and an analysis of the corresponding transcripts revealed the existence of a mechanism of alternative splicing of *JAZ* gene transcripts. Thus, this study suggested that the stress caused transcriptionally and post-transcriptionally by latex harvesting regulates the jasmonate signalling pathway in *Hevea*.

## Methods

### Plant material

For the transcript analyses, plant material of clone PB 260 grew in accordance with the conditions described in Duan and coll. [35]. Samples corresponding to reproductive tissues (immature and mature, male and female flowers, zygotic embryos) were added to this study. Flowers were collected from 15-year-old trees. Zygotic embryos were collected from 5-year-old trees. *In vitro* plantlets of clone PB 260 were obtained by somatic embryogenesis with line CI07060. The plantlets were acclimatized and grown for 3 months in a greenhouse at a temperature of 27°C with 45% humidity. Several treatments mimicking abiotic stress were carried out, such as wounding, methyl jasmonate (MeJA), ethylene (ET) and dehydration at 1, 4, 8 and 24 hours. Drought stress response was controlled by following *HbERFIVa3* (orthologue to DREB2A) transcript accumulation during this stress [49], whereas efficiency of wounding, ethylene and MeJA treatment were controlled using *HbERF-IXc4* and *HbERF-IXc5* (orthologue to ERF1) [37,50]. The bark was wounded every 0.5 cm by scarification with a razor blade. The ET and MeJA treatments were carried out by placing the plants in a 300-l open-door Plexiglas box overnight before treatment. Five parts per million of pure ET gas (1.5 ml/300 l) was injected into the sealed air-tight box. A concentration of 100 μl of liquid ≥95% MeJA solution (Sigma, St Louis, MO) was diluted in 500 μl of absolute ethanol and then placed on Whatmann paper inside the box for gas release. Control plants used for the ET and MeJA treatments were placed in the box and exposed to air only. The dehydration treatment was carried out by taking the plants out of their pots and leaving them to dry under laminar air flow. Each sample was collected 1 h, 4 h, 8 h and 24 h after treatment.

RNA samples were collected and prepared at the CRRC, RRIT, Sanam Chaikhet District, Chachoengsao 24160, Thailand (13.39°N latitude and 101.26°E longitude). These locations and our activities did not require any specific permission. The field studies did not involve endangered or protected species.

### Total RNA extraction

Total RNAs were extracted from one gram of fresh matter using the caesium chloride (CsCl) cushion method adapted from Sambrook and coll. (Sambrook et al. [51]) by Duan and coll. (Duan et al. [37]). DNA contamination was checked by PCR amplification using primers of the actin gene.

### Primer design and transcript abundance analysis by qPCR

Total RNA integrity was checked by electrophoresis. For each candidate gene a blast was performed against the transcriptome of PB 260, to define highly conserved regions. The qPCR primers were designed outside these regions using the Primer 3 module of Geneious Pro software version 5.3.6 (Biomatters Ltd., New Zealand). Each primer pair was blasted against the transcriptome library. qPCR and the fusion curve of the PCR amplicon were done using a mix of cDNAs. In addition, the specificity of each primer pair was checked by sequencing the PCR amplicon. The sequences of the primers used are listed in Additional file 1.

cDNAs were synthesized from 2 μg of total RNA to a final reaction volume of 20 μl using a Revert AidTM M-MuLV Reverse Transcriptase (RT) kit according to the manufacturer’s instructions (MBI, Fermentas, Canada). Full-length cDNA synthesis was checked for each sample by PCR amplification of the actin. Quantitative gene expression analysis was carried out by qPCR using a Light Cycler 480 (Roche, Switzerland) as described by Putranto and coll. [52]. Real-time PCR was carried out for eleven housekeeping genes in order to select the most stable gene as the internal control for all the compared tissues (HbelF1Aa, HbUBC4, HbUBC2b, HbYLS8, HbRH2b, HbRH8, HbUBC2a, HbalphaTub, Hb40S, HbUbi, HbActin). HbRH2b was selected as the best reference gene due to its stability in the different tested tissues [39] (Additional files 2 and 3). The transcript abundance of each gene was relatively quantified by normalization relative to the transcript abundance of the *HbRH2b* reference gene. All the normalized ratios corresponding to transcript accumulation were calculated automatically by Light Cycler software version 1.5.0 provided by the manufacturer. Heatmap representation was carried out on normalized and centered ΔCt values, using the heatmap2 function of the R software gplots package [53,54].

### Statistical analyses

qPCR reactions were carried out with 3 biological replicates. The statistical analyses were ANOVAs carried out on raw data after logarithmic transformation. An ad-hoc Tukey HSD (Honestly Significantly Different) test was carried out for the analysis of transcripts in the different organs and different clones. Values with the same letter were not significantly different. For the analysis of transcripts in response to abiotic stress and to hormone treatments, a comparison of means test (Student or Wilcoxon test, depending on the data) was carried out between the control and the treatment at each point of the kinetics. If, at one point, at least one significant difference was found an “s” was indicated, otherwise “ns” was indicated”.

### Phylogenetic analysis of *JAZ* genes

Multiple alignment was carried out on the total protein sequences of the *JAZs* of *Arabidopsis* and *Hevea*. Alignment with Gblocks software [55] led to the isolation of conserved blocks at least 10 amino acids long. The blocks were then used to construct the phylogenetic tree using PhyML software [56] which implements a maximum likelihood tree reconstruction method using the LG + gamma model, starting from a BioNJ tree [57]. A RAP-Green analysis was carried out using the original tree from PhyML to predict duplications [58]. The final tree was visualized with the Archaeoptheryx program [59].

### Test of transcriptional activity by transient expression in BY-2 cells of tobacco

Transactivation experiments were carried out following the procedure described by Chaabouni and coll. [60]. The *pJAZ_1405(−267)* and *pJAZ_1405(−955)* promoters were cloned to the pMDC107 vector [61], thereby controlling the GFP reporter gene. The *MYC_771* and *MYC _94937* genes were cloned under control of the 35S promoter to the pMDC32 vector. Transactivation assay was carried with several controls. First, a window excluding debris and define auto-fluorescence was defined from protoplast solution without transformation. Second, a negative control was obtained by protoplast transformation with the empty reporter vector and effector vector. Third, protoplast transformation with two vectors pJAZ and pHbMYC was carried out. The negative control is used as reference for the calculation of transactivation activity as follows: (pJAZ_1405(−955) + pHbMYC)/(pJAZ_1405(−267) + pHbMYC). This method avoids any risk of disturbance due to the gateway cassette present in pMDC32 vector. The primer sequences used for gateway cloning are listed in Additional file 4. Transformation was replicated 6 times independently. After 16 h, GFP expression was quantified by flow cytometry (FACS Calibur II instrument, BD Biosciences) on the Montpellier Rio Imaging (MRI) platform. Data were analysed using Flowing Software version 2.5.0.

### Accession numbers

Reads from this library are those published by Duan and coll. (Accession: PRJNA235297 ID: 235297) available on NCBI database.

## Results

### Identification of the different genes acting in the jasmonate signalling pathway

The different genes acting in the jasmonate signalling pathway were identified from the “comprehensive transcriptome” published by Duan and coll. [35] using TBLASTN with the corresponding *Arabidopsis* sequences as the query. For each gene, we selected the one that came out with the best score. For each gene identified in that way, we checked for the conservation of the domains characteristic of each family using INTERPROSCAN. We thus identified 6 contigs corresponding to *JAR*, 2 for *COI*, 10 for *JAZ* and 3 for *MYC*. The *JARs* belonged to the GH3 multigene family [62]. GH3s are generally characterized by the presence of 3 small conserved motifs [63]. Motif III was found to be highly conserved in the 6 *Hevea* sequences. Motifs I and II were found in JAR_5108, JAR_14894 and JAR_59958, while JAR_20347 and JAR_20244 did not display motif I. The absence of motif I in the N terminal part of the proteins deduced from the *JAR_20347, JAR_21367* and *JAR_20244* contigs was linked to the fact that the contig sequences were incomplete at 5′ (Additional file 5). An analysis of the protein sequences revealed that the two HbCOI, HbCOI_2304 and HbCOI_3058*,* were characterized by the presence of an F-box domain, along with 3 leucine-rich repeat (LRR) domains (Additional file 6). All of the JAZ proteins identified had the 2 characteristic domains, TIFY and Jas (Additional file 7) [64]. HbMYC_424, HbMYC_771 and HbMYC_94937 all had a bHLH DNA binding domain, along with the 4 conserved regions (I to IV) (Additional file 8) [65]. An analysis of the transcriptome of rubber clone PB260 also enabled us to identify the partners *HbNINJA_6328* (Additional file 9) and *HbTPL_7591* (Additional file 10). HbNINJA displayed 46% of identical residues with At4g28910, while HbTPL displayed very strong homology with At1g15750 as 85% of the amino acids were identical. We were also able to identify an orthologue of AtMED25, HbMED25_16787, which displayed 50% identity (Additional file 11).

### Expression profile of the different genes acting in different developing tissues

**Figure 1:**
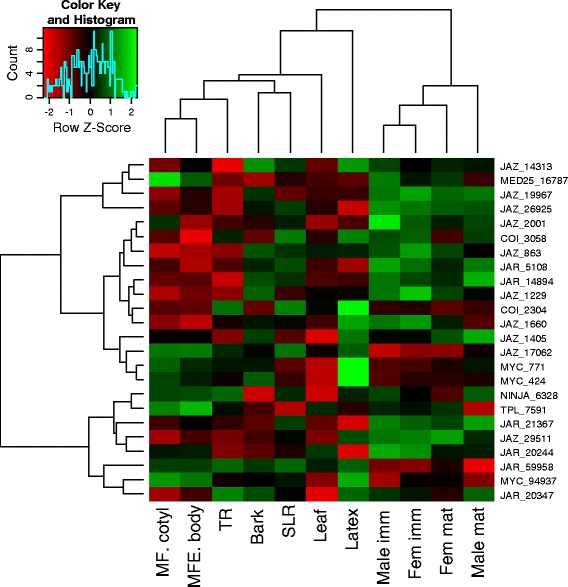
**Heatmap representation of the expression of genes acting in the jasmonate pathway in different rubber tree tissues.** The data obtained by quantitative RT-PCR corresponded to the levels of the actor transcripts in total RNA samples extracted from mature fruit cotyledon (MF. cotyl), mature fruit embryo bodies (MFE.body), taproots (TR), bark, secondary lateral roots (SLR), leaves, latex, immature male flowers (Male imm), immature female flowers (Fem imm), mature female flowers (Fem mat), and mature male flowers (Male mat). The data presented correspond to averages of 3 independent biological replicates. The red and green colours correspond to low and high gene expression, respectively. The heat map was generated using R software.

In order to characterize the JA signalling pathway in different *Hevea* tissues, the transcript abundance of all the genes identified was analysed by qPCR in zygotic embryos (embryo body and cotyledon), roots (TR: taproot and SLR:secondary lateral root), bark, leaves, latex, and male and female flowers at the immature and mature stages (Figure 1). Heatmap representation combined with an analysis of variance (ANOVA) was used to class the genes according to their level of expression in each tissue (Additional file 12). In addition, hierarchical clustering was used to identify tissues that displayed the same transcriptional signature. The different parts of the embryo displayed the same transcriptional landscape. Overall the jasmonate-related genes implicated in flowers were those that were not expressed in the embryo. Male and female flowers, whether mature or immature, were grouped together, suggesting that the same jasmonate-related genes were involved, whatever the flower gender and development stage. The transcriptional signature in the taproot (TR) was grouped with the embryo, while most of the genes expressed in the secondary lateral roots (SLR) were also expressed in bark. Surprisingly, latex displayed an original profile, with a large number of over-expressed signalling genes (*JAZ_14313, COI_3058, COI_2304, JAZ_1660, JAZ_1405, JAZ_17062, MYC_771, MYC_424, MYC_85795, MYC_94937*). These results confirmed the importance of the jasmonate pathway in latex. In addition, it was interesting to see that *TPL_7591* and *NINJA_6328*, which, *a priori* function together, were in the same cluster. *MED25_16787* had an expression profile similar to that of *JAZ_14313*.

### Regulationby abiotic stress of genes acting in the jasmonate pathway

**Figure 2:**
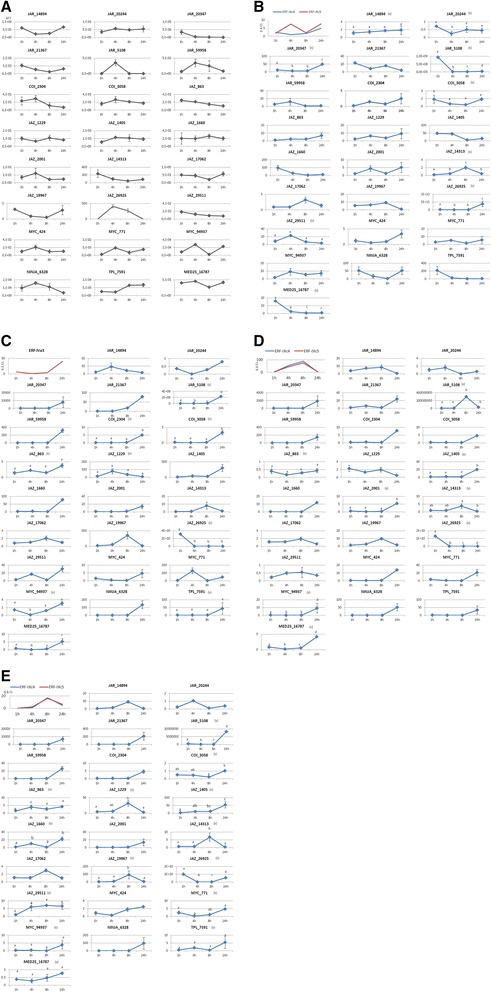
**Expression pattern of the jasmonate transduction pathway members in** ***Hevea*** **plantlets in response to abiotic and hormonal stress.** The data obtained by quantitative RT-PCR corresponded to the transcript accumulation of genes acting in the JA signalling pathway in response to wounding, dehydration, ethylene and methyl jasmonate (MeJA) in stems of 3-month-old plantlets. RNAs were collected after 1 h, 4 h, 8 h and 24 h of treatment.,Gene expression values were normalized using Rh2B as internal control, and then, ratio between treated and untreated plant have been calculatedfor each time point. A comparison of means test was carried out to compare the transcript abundance in stressed plants and control plants. Significant changes were indicated by “s” above error bars. **(A)** Expression pattern of jasmonate pathway genes in control plant. **(B)** Expression pattern of jasmonate pathway genes in response to wounding. Expression pattern of *HbERFIXc4*and *HbERFIXc5* have been tested to validate efficiency of wounding treatment. **(C)** Expression pattern of jasmonate pathway genes in response to dehydration. Expression pattern of *HbERFIV-a3* has been tested to validate efficiency dehydration treatment. **(D)** Expression pattern of jasmonate pathway genes in response to ethylene. Expression pattern of *HbERFIXc4*and *HbERFIXc5* have been tested to validate efficiency ethylene treatment. **(E) **Expression pattern of jasmonate pathway genes in response to jasmonate. Expression pattern of *HbERFIXc4*and *HbERFIXc5* have been followed to validate efficiency jasmonate treatment. The data presented correspond to the meanof 3 independent biological replicates.

The relative transcript abundance was monitored over time for the 24 contigs corresponding to the genes of the jasmonate signalling pathway in response to wounding, dehydration, ethylene and methyl jasmonate (MeJA) (Figure 2). In the aim to validate efficiency of treatments, *Hevea* orthologous of *Arabidopsis* genes, known to be regulated by wounding, dehydration, ethylene and MeJA were used as positive control. Orthologous to DREB2 and ERF1 were identified in *Hevea* by Piyatrakul and coll. [37]. Expression of the *DREB2* genes is induced by drought stress and is involved in drought-responsive gene expression [49]. HbERF-IVa3 is an orthologous of DREB2A and DREB2B [37]. ERF1 is induced by various stresses including wounding and hormonal treatments, acts at the crosstalk of jasmonate and ethylene signalling pathways. Its two putative orthologous genes in Hevea, HbERF-IXc4 and HbERF-IXc5 [37], were used to validate wounding, ethylene and jasmonate treatments in this study. The transcript abundance of all the genes of the jasmonate signalling pathway was modified between 1 h and 24 h, whatever the treatment. The transcripts of *JAR_14894, JAR_20347, JAR_5108* accumulated significantly in response to wounding, while the transcript abundance of *JAR_20244* was significantly lower than in the control (Figure 2A,B). Only the transcript of *JAR_5108* accumulated significantly in response to dehydration. For each type of stress, at least one transcript of each partner of the COI-JAZ-MYC module was highly activated (Figure 2C). Interestingly, several genes acting in the signalling pathway were also regulated by ethylene (Figure 2D). Statistical analyses showed that *JAR_5108, JAZ_863, JAZ_1405, JAZ_2001, JAZ_14313, JAZ_26925* and *MYC_94937* were regulated by ethylene. It should be noted that, among the *JAR* and *COI*, only the transcripts of *JAR_5108* and *COI_3058* were significantly regulated by JA (Figure 2E). Of the 10 *JAZ* genes studied, the transcripts of eight of them were significantly accumulated in response to JA (*JAZ_863, JAZ_1229, JAZ_1405, JAZ_1660, JAZ_14313, JAZ_19967, JAZ_26925, JAZ_29511*). The transcripts of *MYC_771* and *MYC_94937* were significantly accumulated in response to JA. *TPL_7591, NINJA 6328* and *MED25_16787* were regulated overall in the same manner. In response to wounding, their transcript abundance increased rapidly in 1 h, while in response to ET and MeJA, they only accumulated after 24 h. Taken together, these results suggest that the genes involved in JA signalling are expressed in response to abiotic stress, but it was not always the same JARs, COIs, JAZs and MYCs that were involved depending on the stress in that signalling.

### Genotypic regulation of genes involved in the JA pathway in response to harvesting stress in mature trees

**Figure 3:**
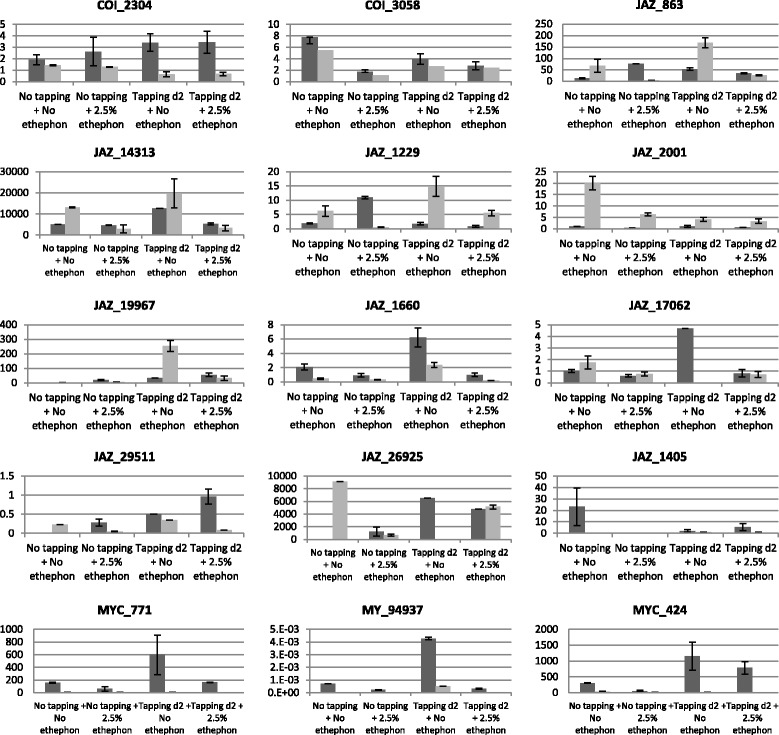
**Regulation of COI-JAZ-MYC module members in** ***Hevea*** **in response to harvesting stress in latex and in bark.** The accumulation of each COI-JAZ-MYC member was studied in the latex (dark grey bar) and bark (light grey bar) of adult trees, during latex production. During latex production the trees were tapped (3,4) every 2 days, or not (1,2), and stimulated by 2.5% ethephon, once per month (2,4), or not (1,3). The data presented correspond to averages of 3 independent biological replicates.

The transcript abundance of genes belonging to the COI-JAZ-MYC module were studied in the latex and bark of tapped rubber trees (Figure 3, Additional file 13). During harvesting, the transcripts of the COI-JAZ-MYC module were regulated in both latex and bark. Our results showed that the expression level for *COI_2304* was significantly higher in latex than in bark (p < 0.01). In latex, the transcripts of *COI_2304* were accumulated in response to tapping, while in bark the transcript abundance decreased in response to that stress (p < 0.01). Treatment with ethephon tended to reduce the transcript abundance of *COI_3058* (p < 0.01) in latex and in bark. The results suggested that the transcript regulation of *JAZ* genes was specific to each gene and that no general tendency could be brought out for the family as a whole. For example, *JAZ_1229, JAZ_2001* and *JAZ_19967* were significantly more expressed in bark than in latex, while the reverse was true for *JAZ_1405, JAZ_1660, JAZ_17062* and *JAZ_29511*. In latex, *JAZ_1405* was the only *JAZ* to be significantly repressed by tapping (p < 0.01) (Additional file 13), while *JAZ_863* (p < 0.01), *JAZ_1660* (p < 0.01), *JAZ_17062* (p < 0.01), *JAZ_29511* (p < 0.01) and *JAZ_26925* (p < 0.01) were induced by tapping when there was no ethephon treatment. Interestingly, the transcripts of *JAZ_17062* and *JAZ_26925* were accumulated in an opposite manner in latex and bark in response to tapping. In bark, *JAZ_863* (p < 0.01), *JAZ_1229* (p < 0.01), *JAZ_19967* (p < 0.01), *JAZ_1660* (p < 0.01), and *JAZ_29511* (p < 0.01) were induced by tapping, while the expression of *JAZ_2001* was repressed by tapping (p < 0.01). In the majority of cases, ethephon reduced *JAZ* transcript abundance. However, in latex, the transcripts of *JAZ_29511* and *JAZ_863* were accumulated in the presence of ethephon (p < 0.01 and p < 0.01, respectively). In bark, when the tree was tapped, the transcripts of *JAZ_26925* were accumulated in the presence of ethephon (p < 0.01). The 3 MYC factors tested displayed the same expression profile. In fact, they were expressed more in latex than in bark and were strongly induced by tapping. Together, these results suggested that JA played a role during latex harvesting.

### Clonal regulation of genes acting in the jasmonate signalling pathway

**Figure 4:**
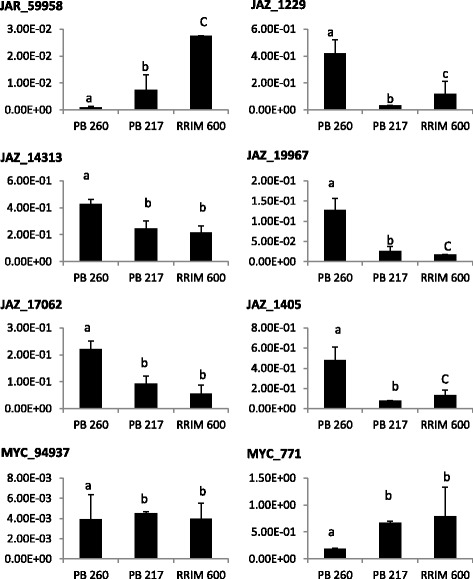
**Expression of genes acting in the jasmonate pathway in 3** ***Hevea*** **clones.** The transcript abundance of the 24 identified genes was studied by qPCR on latex from PB 260, INC 53 and RRIM 600. According to an analysis of variance 8 transcripts were significantly over-represented in one or two clones (Additional file 14). The data presented correspond to averages of 3 independent biological replicates. Different letters correspond to significantly different values after theTukey HSD test (p < 0.05).

In order to find out whether the jasmonate signalling pathway might be involved in the differences in laticifer metabolism observed at clonal level, we studied the transcript abundance of each of the genes acting in the pathway in clones PB260, INC53 and RRIM600. Of the 24 genes studied, only 8 displayed a transcript abundance that was significantly different between clones (Figure 4, Additional file 14). *JAR_59958* and *MYC_771*, positive regulators of the JA signalling pathway, were highly expressed in INC53 and RRIM600. On the other hand, the transcripts of the *JAZ* negative regulators were more expressed in clone PB260. These results showed that the positive regulators of the JA pathway are preferentially accumulated in clones with a slow laticifer metabolism, while the negative regulators are accumulated more in clones with a rapid laticifer metabolism.

### Conservation of the structure *HbJAZ* genes and that of their *Arabidopsis* orthologue

**Figure 5:**
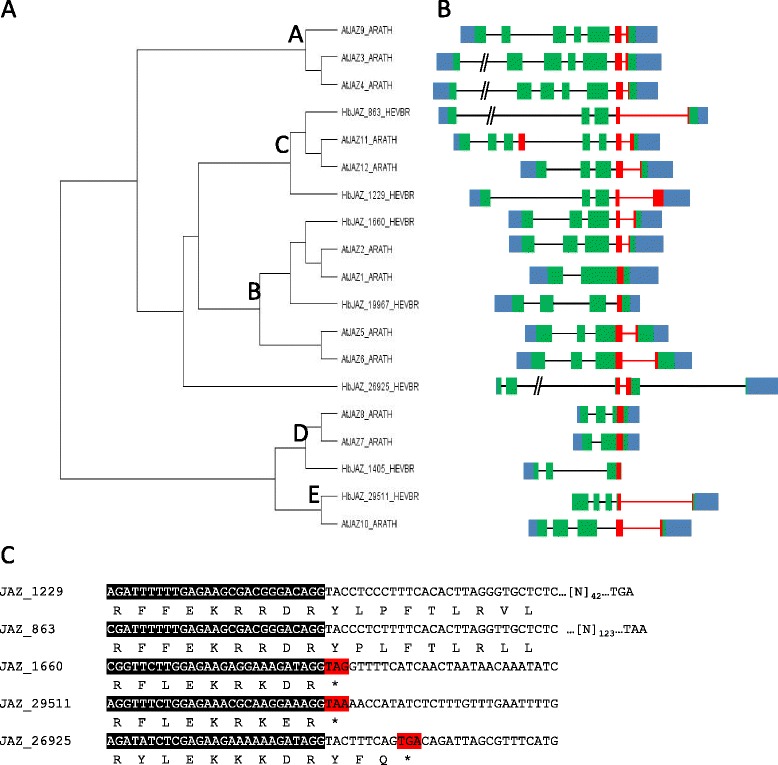
**HbJAZ gene structure is conserved with the** ***Arabidopsis*** **orthologue. (A)** Phylogenetic tree constructed from the amino acid sequence of 12 full-length AtJAZs and 7 full-length HbJAZ, showing five **(A–E)** subclades of proteins. **(B)** Intron/exon organization of the corresponding genes. Thick green and blue bars indicate coding regions and non-coding untranslated regions in exons, respectively. The Jas motif coding region is depicted in red. Introns are depicted by a thin black horizontal line or, in the case of the Jas intron, a red line. **(C)** Sequence of the Jas exon/intron junction in five *HeveaJAZ* genes containing the intron. Exon sequences are highlighted in black with the predicted amino acid sequence. Illustration adapted from [26].

A study of the phylogeny and structure of the *JAZs* of *Hevea* was carried out on 7 *JAZs* (*JAZ_1660, JAZ_19967, JAZ_1405, JAZ_29511, JAZ_26925, JAZ_863, JAZ_1229*) which were of interest because they are regulated both by JA and by tapping. A phylogenetic analysis based on the protein sequences of the HbJAZ sand AtJAZs revealed an organization primarily in three sub-classes B, C and D previously defined by Chung [26], with sub-class A not being represented by any *JAZ* member of *Hevea* (Figure 5A). AtJAZ10, previously assigned to sub-class C, revealed stronger homology with HbJAZ_29511 to form a new sub-class that we called sub-class E. HbJAZ_26925 was not grouped in the previously described sub-classes and formed a new sub-class. The gene structure of the 7 *JAZs* of interest was analysed using the scaffold sequences corresponding to the genome of rubber clone CATAS 7-33-97 (Figure 5B). Most rubber tree *JAZs* possess an intron in the Jas domain, which is similar to what has been described in *Arabidopsis*. The sequence analysis predicted that retention of the 5′ region of the Jas intron splicing site during pre-mRNA processing of *JAZ_1660*, *JAZ_29511* and *JAZ_26925* generated a premature termination codon immediately or very rapidly (Figure 5C). By using both primers overlapping the splicing zone and primers inside the intron, it was possible to confirm alternative splicing by sequencing of the qPCR amplicons for *JAZ_1229, JAZ_863, JAZ_1660, JAZ_29511* and *JAZ_26925*.

### Alternative splicing of *HbJAZ* is regulated by tapping stress

**Figure 6:**
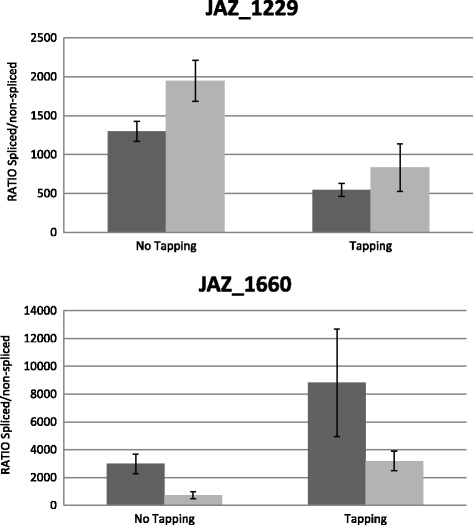
**Regulation of alternative splicing by tapping and ethephon treatment inPB260 clone.** The data presented correspond to the ΔCt spliced form/ΔCtunspliced form ratio in latex. Ratios were studied under four conditions, untapped and tapped (left and right bars, respectively), stimulated or not by 2.5% ethephon (grey and black bars, respectively). The data presented correspond to averages of 3 independent biological replicates.

In order to gain a clearer understanding of *JAZ* post-transcriptional regulation in response to tapping stress in rubber trees, we used qPCR to measure the spliced form/non-spliced form ratio of transcripts in the latex and bark of tapped or untapped rubber trees, treated or not with ethephon (Figure 6, Additional file 15). Despite the existence of non-spliced forms for the transcripts of *JAZ_29511, JAZ_863* and *JAZ_26925*, this was not detected by qPCR in the samples tested. The alternative splicing of *JAZ_1229* and *JAZ_1660* was significantly regulated by tapping and by ethephon treatment. Our results suggested opposite regulation between the splicing of *JAZ_1229* and *JAZ_1660*. In fact, tapping induced the non-spliced form of *JAZ_1229*, suggesting an increase in the repressive form of that *JAZ*, while the same stress induced the spliced form of *JAZ_1660*.

### MYC_771 and MYC_94937 regulate the transcription of *JAZ_1405*

**Figure 7:**
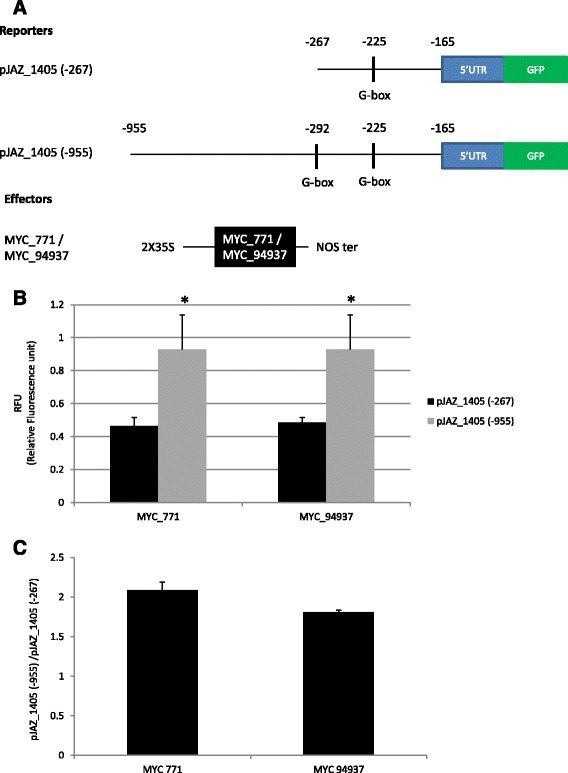
**MYC_771 and MYC_94937 regulate transcription of** ***JAZ_1405.*** Transient expression in a single cell system was used to assess the transcriptional effect of MYC_771 and MYC_94937 proteins on *JAZ_1405*. The fluorescence of the reporter gene was measured by flow cytometry upon co-transfection with a reporter construct (*pJAZ_1405*::GFP) and an effector construct (35S::HbMYC_771 or 35S::HbMYC94937). The basal fluorescence obtained in the assay transfected with the reporter construct and *pJAZ_1405(−267)* was taken as the reference (100% relative fluorescence). **(A)** Schematic representation of the constructs used for transient expression assays. The reporter construct consisted of the *JAZ_1405* promoter and GFP coding sequence. The effector constructs expressed HbMYC_771 and HbMYC_94937 under the control of the CaMV 35S promoter. **(B)**The fluorescence of protoplasts co-transformed with pJAZ_1405::GFP and pMDC32 were used as reference (RFU = 1). Ratio of fluorescence from *pJAZ_1405 (−267)*::GFP or pJAZ_1405(−955)::GFP with HbMYC_771 or HbMYC_94937 on the reference fluorescence were displayed in RFU. Star indicate a significant difference of fluorescence obtained with the same MYC for long and short promoters. **(C)** Effect of MYC_771 and MYC_94937 on the *JAZ_1405* promoter. The value with the short promoter was set at 1 and relative values are shown. Error bars indicate the standard deviation of results from 6 replicates.

The transcripts of certain *JAZs* were not subjected to alternative splicing like *JAZ_1405*. Hierarchical clustering grouped *MYC_771, MYC_424, JAZ_1405* and *JAZ_17062* in the same cluster. Given that the transcripts of *MYC_424* and *JAZ_17062* were not significantly regulated by jasmonate, we focused on the transcriptional regulation of *JAZ_1405* by MYC_771 and MYC_94937 which, in response to JA, displayed the same expression profile. To that end, we cloned 2 versions of the *JAZ_1405*promoter (Figure 7A): a short sequence (*pJAZ_1405(−267)*) which contained 267 bp upstream of the ATG and which contained a G-box, known to be regulated by MYC factors [16] and a longer sequence (*pJAZ_1405 (−955)*) with a size of 955 bp upstream of the ATG and which contained 2 G-boxes. The GFP reporter gene under the control of promoters*pJAZ_1405 (−267)* or *pJAZ_1405 (−955)*was co-transformed with 35S ::HbMYC_771 or 35S ::HbMYC_94937 (Figure 7A,B). The results showed that the 2 *MYCs* had a transcriptional activity that was twice as strong with the *pJAZ_1405(−955)* promoter than with the *pJAZ_1405(−267)* promoter (Figure 7C,).

## Discussion

### Laticifer cells display a transcript pattern for genes acting in the jasmonate pathway that differs from the other tissues

To date, data regarding the jasmonate signalling pathway have been limited in latex plants to *Hevea*. Although a few genes acting in the jasmonate signalling pathway (*COI1, JAZ, MYC*) have been identified in *Hevea* [40-42], our study led to the identification of all the factors (6 *JAR*, 2 *COI*, 10 *JAZ*, 3 *MYC*, 1 *NINJA*, 1 *TPL*, 1 *MED25*). Recently, a sequence of *HbCOI1* was isolated from clone RRIM600 (EU136026) [40]. Our analyses enabled us to identify an allele of this gene in clonePB260, *HbCOI_2304. HbCOI_2304* displayed 95% identity with the sequence of RRIM600. So far, in *Hevea brasiliensis*, only one *JAZ* sequence has been identified (*HbJAZ1* : GQ369508) in clone RY-7-33-97 [41]. An analysis of the transcriptome of clone PB260 resulted in identification of the *HbJAZ_1660* sequence, which displayed 100% homology with *HbJAZ1.* In addition, the same laboratory isolated 2 transcription factors, *HbMYC*1 and*HbMYC2* (*HblMYC1* :GU434304 and *HblMYC2* : HM061097) in clone RY-7-33-97, which have been described as orthologous to *AtMYC2* [42]. An analysis of the transcriptome led to the identification of these 2 genes. *MYC_424* displayed 99% identity with *HbMYC1*, like *MYC_771* orthologous to *HbMYC2.* The number of *COI* and *JAZ* gene models is similar in *Hevea,* rice, *Arabidopsis*, poplar, grapevine and *Brachypodium*, but the number of *JARs* is larger in *Hevea* (6) than the one or two genes found in the other species. However, given the diversity of GH3s, it is highly likely that not all of them are involved in JA conjugation with Ile (Table 1).

**Table 1:** **Number of identified members of JAR, COI and JAZ genes in the rubber tree, rice,** ***Arabidopsis*** **, poplar, grapevine and** ***Brachypodium***

**Genes**	**Contigs**	**Number of genes (data from ** **http://rice.plantbiology.msu.edu** **)**				
		***Rice***	***Arabidopsis***	***Poplar***	***Grapevine***	***Brachypodium***
**JAR**	**6**	**1**	**1**	**2**	**1**	**1**
**COI**	**2**	**2**	**1**	**2**	**1**	**2**
**JAZ**	**10**	**15**	**12**	**14**	**9**	**11**

Laticifers cells displayed an original signature of transcripts for the JA signalling pathway since they stood out from all the other tissues tested (Figure 1). Transcript analyses, obtained on PB260, confirmed the results published by Peng and coll. on RRIM 600 [40] showing that *HbCOI_2304* is preferentially expressed in latex. In addition, *HbCOI_3058* displayed a similar profile (Figure 1). In clone RY-7-33-97, Zhao and coll. showed that the transcripts of *HbMYC1* and *HbMYC2* were preferentially accumulated in latex [42]. Our results, obtained on clone PB 260 confirmed that tendency (Figures 1 and 2). However, our results did not confirm an abundance of *MYC_424* transcripts in response to JA observed in clone RY-7-33-97 [42] (Figure 2E). To date, no study has shown the potential implication of *JAZs* in the laticifer metabolism. An analysis of the *HbJAZ* expression profile showed that the transcripts of 5 *HbJAZs* were more highly accumulated in latex than in the other tissues. For instance, in addition to the transcripts of *COI*, *JAZ* and *MYC*, the transcripts of the *JAR_59958* and *JAR_20347* genes were highly accumulated in this tissue. All these results suggested that the jasmonate signalling pathway in laticifer cells involved specific actors. This confirmed the importance of studying this pathway in latex production but also in response to harvesting stress.

### Harvesting stress can take both common and distinct wounding and dehydration pathways

Latex harvesting amounts to strong abiotic stress that can lead to a physiological syndrome such as TPD. A study of transcript abundance in response to wounding, dehydration, and to hormonal treatments, ethylene and jasmonate, helped to determine what types of abiotic stress and signalling pathways occurred during tapping. The jasmonate signalling pathway was regulated by all the types of stress tested, suggesting the involvement of that pathway in the response to each of the types of stress. Although the comparison was difficult, as the tissues and times tested were not exactly the same, our results confirmed that the transcript abundance of *HbMYC1* and *HbMYC2*, corresponding to *MYC_424* and *MYC_771,* decreased after 6 h of MeJA treatment, then rose from 24 h onwards [42] (Figure 2E). The results suggested that the signalling pathways of abiotic and harvesting stress may activate the same genes. Indeed, *JAZ_863,* repressed by ethylene in the stem and by ethephon in bark, was at the same time induced by dehydration and tapping (Figures 2D and 3). In addition, *JAZ_29511* and *JAZ_14313* were induced by both wounding and tapping, while *JAZ_1229* was induced by dehydration and tapping (Figures 2 and 3). Despite that, the results in this study suggested that some independent wounding, dehydration and ethylene pathways were activated during latex harvesting. Indeed, *JAZ_2001, JAZ_14313* and *JAZ_26925* were induced by ethylene at 24 h, 8 h and 1 h respectively, but were repressed by ethephon in bark (Figures 2 and 3). In latex, the transcripts of *COI_2304, MYC_424* and *MYC_771* were accumulated in response to tapping (Figure 3), which confirmed the results in the literature [40,42]. Nevertheless, JAZ members are functionally redundant to some extent [17-19] and the diversity in their structures suggests specific roles for some of them. This hypothesis is corroborated by the diverse expression patterns displayed by HbJAZ in response to various types of abiotic stress.

### HbMYC_771 and MYC_94937 can regulate the *HbJAZ* genes containing a G-box type *cis*-element

In addition to negative regulation of JAZs on MYCs, it has been shown that there exists a positive regulation of *JAZ* transcription by MYCs *via* the G-box *cis*-element [16]. Hierarchical clustering grouped *MYC_771, MYC_424, JAZ_1405* and *JAZ_17062* in the same cluster (Figure 1). Given that the transcripts of *MYC_424* and *JAZ_17062* were not significantly regulated by jasmonate, we focused on the regulation of *JAZ_1405* by *MYC_771* and *MYC_94937* which, in response to JA, displayed the same expression profile (Figure 2). Transactivation experiments showed that the 2 MYC factors tested were able to regulate the transcription of *JAZ_1405* (Figure 7). It is highly likely that this activation of *JAZ_1405* by MYC factors occurs *via* the G-box present in the promoter of that gene. Among the promoters isolated from scaffold sequences, we were able to show that the promoters of *HbJAZ_1229* and *HbJAZ_19967* also possessed a G-box, suggesting transcriptional regulation of these 2 *JAZs* by MYCs. This last result may explain the grouping of *MYC_771* and *JAZ_1405* in the same cluster, but also a similar expression profile for *MYC_94937* and *JAZ_1405* in response to JA.

### *JAZ_1229* and *JAZ_1660* are regulated by alternative splicing in the laticifer cells of tapped trees

The regulation of alternative splicing provides flexibility at transcriptome and proteome level, which helps plants adapt to their environment [66]. However, while the misregulation of alternative splicing has been associated with many human diseases, its biological relevance in plant systems is just beginning to be decipher [67]. Many studies have shown the importance of AS and splicing factors (SF) in the response to abiotic stress (for review, [28]). With the alternative splicing of JAZ proteins, recent studies suggest that it makes it possible to establish a negative feedback loop to attenuate the response to JA in the event of over-stimulation of the signalling pathway [26,27]. To date, this regulation of *JAZs* has never been linked to the response to abiotic stress. An analysis of the structure of *HbJAZs*, along with the sequencing results, confirmed the existence of alternative splicing of the *HbJAZ_1229* and *HbJAZ_1660* genes (Figure 5)*.* An analysis of the exon/intron junction of these 2 *HbJAZs* showed that this splicing led to the introduction of a premature termination codon (PTC). In *Arabidopsis*, the introduction of that PTC leads to the production of a JAZ protein, whose jas domain is absent [26]. In general, the introduction of a PTC engages the transcript in the nonsense-mediated decay (NMD) pathway to degrade it [68,69]. According to the literature, these results suggest that truncated forms of *HbJAZ_1229* and *HbJAZ_1660* are dominant negative regulators [26,27,70]. For the first time, our results brought out a link between the regulation of *JAZ* alternative splicing and abiotic stress. Indeed, our results showed that alternative splicing of *JAZ_1229* and *JAZ_1660* could be regulated by tapping in latex. Curiously, that regulation was opposite for the transcripts of *JAZ_1229* and *JAZ_1660* (Figure 6). An analysis of the abundance of the 2 forms of transcripts of these 2 *HbJAZs* showed that the transcript abundance of the spliced form remained at least 500 times greater than the non-spliced form (Figure 6). This final observation tallied with what is generally observed in *Arabidopsis* since, although intron retention is the most common form of AS (~40%), many of these transcripts are not very abundant at all [71]. Taken together these results suggested a compensatory phenomenon and functional redundancy of *HbJAZ_1229* and *HbJAZ_1660*, which is all the more likely in that they were classed in the same cluster (Figure 1).

### Genes acting in the JA pathway are expressed differentially in the latex of different *Hevea* clones

The results presented here show that the JA signalling pathway was regulated differently depending on the clones tested. Indeed, the negative *JAZ* regulators were over-expressed in PB260, while the transcripts of MYC factors and the JAR enzyme, which are positive regulators of the JA pathway, were accumulated preferentially in clones INC 53 and RRIM600 (Figure 4). These results suggested activation of the JA target genes in clones with a slow laticifer metabolism, more resistant to TPD.

The identification of signalling pathways involved in TPD resistance should make it possible to isolate the master regulators controlling resistance to this syndrome. The identification of JA transduction pathway members and knowledge of their expression pattern open up new ways of improving the TPD-tolerance of *Hevea* clones. The JA signalling pathway is very widely distributed and very highly conserved during evolution. However, a study on extra floral nectar (EFN) excretions by central American acacias revealed that resistance to biotic stress *via* the jasmonate pathway might be induced or constitutive in some phylogenetically very close species [72]. Thus, that study suggests that specific variability of the JA pathway may have some major consequences for resistance to stress.

## Conclusion

The present study provides some molecular clues on how the Jasmonate pathway can be involved in harvesting stress in *Hevea brasilliensis* through (i) the specific expression pattern of jasmonate pathway actors in latex, (ii) their transcriptional regulation in response to harvesting stress, (iii) differential expression depending on the *Hevea* clone, (iv) their putative alternative splicing regulation in response to harvesting. A better understanding of transcriptional regulations during harvesting stress, along with the use of clonal diversity in response to such stress, are therefore a major challenge for improving resistance to stress and rubber production in *Hevea*.

### Availability of supporting data

The authors confirm that all data underlying the findings are fully available without restriction. Character Matrix and phylogenetic tree have been deposited in treebase (ID: 16569) and data are available at the following URL: http://purl.org/phylo/treebase/phylows/study/TB2:S16569.

## Additional files

Additional file 1:
**Sequences of the primers used for qPCR analysis.**
Additional file 2:
**Comparison of Cp values, standard deviation and coefficient of variance for gene expression analysis by real-time RT-PCR of 11 housekeeping genes in in abiotic stress conditions.**
Additional file 3:
**Comparison of Cp values, standard deviation and coefficient of variance for gene expression analysis by real-time RT-PCR of 11 housekeeping genes in harvesting stress conditions.**
Additional file 4:
**Sequences of the primers used for cloning of** ***MYC_771, MYC_94937*** **and** ***pJAZ_1405.***
Additional file 5:
**Amino acids sequence alignment of HbJAR1 with AtJAR1.**
Additional file 6:
**Amino acids sequence alignment of HbCOI with AtCOI.**
Additional file 7:
**Amino acids sequence alignment of HbJAZ with AtJAZ1.**
Additional file 8:
**Amino acids sequence alignment of HbMYC with AtMYC2.**
Additional file 9:
**Amino acids sequence alignment of HbNINJA with AtNINJA.**
Additional file 10:
**Amino acids sequence alignment of HbTPL with AtTPL.**
Additional file 11:
**Amino acids sequence alignment of HbMED25 with AtMED25.**
Additional file 12:
**Analysis of variance of gene expression according the tested tissues.**
Additional file 13:
**Analysis of variance (ANOVA) to test effect of tapping,ethephon treatment, tissue and cross effects on the expression of the different gene of the JA signaling pathway.**
Additional file 14:
**Analysis of variance of gene expression according the tested clone.**
Additional file 15:
**Analysis of variance (ANOVA) to test effect of tapping, ethephon treatment, tree age and cross effect on the ratio spliced/unspliced of JAZ_1229 and JAZ-1660.**

## References

[1] Wasternack C, Hause B (2013). Jasmonates: biosynthesis, perception, signal transduction and action in plant stress response, growth and development: an update to the 2007 review in Annals of Botany. Ann Bot.

[2] De Geyter N, Gholami A, Goormachtig S, Goossens A (2012). Transcriptional machineries in jasmonate-elicited plant secondary metabolism. Trends Plant Sci.

[3] Wolucka B, Goossens A, Inzé D (2005). Methyl jasmonate stimulates the de novo biosynthesis of vitamin C in plant cell suspensions. J Exp Bot.

[4] Sasaki-Sekimoto Y, Taki N, Obayashi T, Aono M, Matsumoto F, Sakurai N, Suzuki H, Hirai MY, Noji M, Saito K, Masuda T, Takamiya K, Shibata D, Ohta H (2005). Coordinated activation of metabolic pathways for antioxidants and defence compounds by jasmonates and their roles in stress tolerance in Arabidopsis. Plant J.

[5] Browse J (2009). The power of mutants for investigating jasmonate biosynthesis and signaling. Phytochemistry.

[6] Staswick P, Tiryaki I, Rowe M (2002). Jasmonate response locus JAR1 and several related Arabidopsis genes encode enzymes of the firefly luciferase superfamily that show activity on jasmonic, salicylic, and indole-3-acetic acids in an assay for adenylation. Plant Cell.

[7] Suza W, Staswick P (2008). The role of JAR1 in Jasmonoyl-L: −isoleucine production during Arabidopsis wound response. Planta.

[8] Fonseca S, Chini A, Hamberg M, Adie B, Porzel A, Kramell R, Miersch O, Wasternack C, Solano R (2009). (+)-7-iso-Jasmonoyl-L-isoleucine is the endogenous bioactive jasmonate. Nat Chem Biol.

[9] Yan J, Zhang C, Gu M, Bai Z, Zhang W, Qi T, Cheng Z, Peng W, Luo H, Nan F, Wang Z, Xie D (2009). The Arabidopsis CORONATINE INSENSITIVE1 protein is a jasmonate receptor. Plant Cell.

[10] Feys BJF, Benedetti C, Penfold C, Turner J (1994). Arabidopsis mutants selected for resistance to the phytotoxin coronatine are male sterile, insensitive to methyl jasmonate, and resistant to a bacterial pathogen. Plant Cell.

[11] Xie D, Feys B, James S, Nieto-Rostro M, Turner J (1998). COI1: an Arabidopsis gene required for jasmonate-regulated defense and fertility. Science.

[12] Xu L, Liu F, Lechner E, Genschik P, Crosby W, Ma H, Peng W, Huang D, Xie D (2002). The SCF(COI1) ubiquitin-ligase complexes are required for jasmonate response in Arabidopsis. Plant Cell.

[13] Ren C, Pan J, Peng W, Genschik P, Hobbie L, Hellmann H, Estelle M, Gao B, Peng J, Sun C, Xie D (2005). Point mutations in Arabidopsis Cullin1 reveal its essential role in jasmonate response. Plant J.

[14] Moon J, Parry G, Estelle M (2004). The ubiquitin-proteasome pathway and plant development. Plant Cell.

[15] Fernández-Calvo P, Chini A, Fernández-Barbero G, Chico JM, Gimenez-Ibanez S, Geerinck J, Eeckhout D, Schweizer F, Godoy M, Franco-Zorrilla JM, Pauwels L, Witters E, Puga MI, Paz-Ares J, Goossens A, Reymond P, De Jaeger G, Solano R (2011). The Arabidopsis bHLH transcription factors MYC3 and MYC4 are targets of JAZ repressors and act additively with MYC2 in the activation of jasmonate responses. Plant Cell.

[16] Figueroa P, Browse J (2012). The Arabidopsis JAZ2 promoter contains a G-Box and thymidine-rich module that are necessary and sufficient for jasmonate-dependent activation by MYC transcription factors and repression by JAZ proteins. Plant Cell Physiol.

[17] Chini A, Fonseca S, Fernández G, Adie B, Chico JM, Lorenzo O, García-Casado G, López-Vidriero I, Lozano FM, Ponce MR, Micol JL, Solano R (2007). The JAZ family of repressors is the missing link in jasmonate signalling. Nature.

[18] Thines B, Katsir L, Melotto M, Niu Y, Mandaokar A, Liu G, Nomura K, He S, Howe G, Browse J (2007). JAZ repressor proteins are targets of the SCF(COI1) complex during jasmonate signalling. Nature.

[19] Yan Y, Stolz S, Chételat A, Reymond P, Pagni M, Dubugnon L, Farmer E (2007). A downstream mediator in the growth repression limb of the jasmonate pathway. Plant Cell.

[20] Chini A, Fonseca S, Chico J, Fernández-Calvo P, Solano R (2009). The ZIM domain mediates homo- and heteromeric interactions between Arabidopsis JAZ proteins. Plant J.

[21] Pauwels L, Goossens A (2011). The JAZ proteins: a crucial interface in the jasmonate signaling cascade. Plant Cell.

[22] Vanholme B, Grunewald W, Bateman A, Kohchi T, Gheysen G (2007). The tify family previously known as ZIM. Trends Plant Sci.

[23] Chung H, Howe G (2009). A critical role for the TIFY motif in repression of jasmonate signaling by a stabilized splice variant of the JASMONATE ZIM-domain protein JAZ10 in Arabidopsis. Plant Cell.

[24] Demianski A, Chung K, Kunkel B (2012). Analysis of Arabidopsis JAZ gene expression during Pseudomonas syringae pathogenesis. Mol Plant Pathol.

[25] Çevik V, Kidd BN, Zhang P, Hill C, Kiddle S, Denby KJ, Holub EB, Cahill DM, Manners JM, Schenk PM, Beynon J, Kazan K (2012). MEDIATOR25 acts as an integrative hub for the regulation of jasmonate-responsive gene expression in Arabidopsis. Plant Physiol.

[26] Chung H, Cooke T, Depew C, Patel L, Ogawa N, Kobayashi Y, Howe G (2010). Alternative splicing expands the repertoire of dominant JAZ repressors of jasmonate signaling. Plant J.

[27] Moreno J, Shyu C, Campos M, Patel L, Chung H, Yao J, He S, Howe G (2013). Negative feedback control of jasmonate signaling by an alternative splice variant of JAZ10. Plant Physiol.

[28] Staiger D, Brown JWS (2013). Alternative splicing at the intersection of biological timing, development, and stress responses. Plant Cell.

[29] Chrestin H, Bangratz J, d’Auzac J, Jacob JL (1984). Role of the lutoidic tonoplast in the senescence and degradation of the laticifers of *Hevea brasiliensis*. Pflanzenphysiol.

[30] Gohet E (1996). La production de latex par Hevea brasiliensis. Relation avec la croissance: influence de différents facteurs: origine clonale, stimulation hormonale, réserves hydrocarbonées. Physiologie végétale et développement.

[31] Jacob J-L, Prévôt J-C, Roussel D, Lacrotte R, Serres E, d’Auzac J, Eschbach J-M, Omont H, Auzac J, Jacob J-L, Chrestin H (1989). Yield limiting factors, latex physiological parameters, latex diagnosis, and clonal typology. Physiology of Rubber Tree Latex.

[32] Sun J-Q, Jiang H-L, Li C-Y (2011). Systemin/Jasmonate-mediated systemic defense signaling in tomato. Mol Plant.

[33] Hao B-Z, Wu J-L (2000). Laticifer differentiation in Hevea brasiliensis: induction by exogenous jasmonic acid and linolenic acid. Ann Bot.

[34] Zeng R, Duan C, Li X, Tian W, Nie Z (2009). Vacuolar-type inorganic pyrophosphatase located on the rubber particle in the latex is an essential enzyme in regulation of the rubber biosynthesis in Hevea brasiliensis. Plant Sci.

[35] Duan C, Argout X, Gébelin V, Summo M, Dufayard JF, Leclercq J, Kuswanhadi ᅟ, Piyatrakul P, Pirrello J, Rio M, Champion A, Montoro P: **Identification of the hevea brasiliensis AP2/ERF superfamily by RNA sequencing.** *BMC Genomics* 2013, **14:**30. 10.1186/1471-2164-14-30 PMC3644242 23324139

[36] Duan C, Rio M, Leclercq J, Bonnot F, Oliver G, Montoro P (2010). Gene expression pattern in response to wounding, methyl jasmonate and ethylene in the bark of Hevea brasiliensis. Tree Physiol.

[37] Piyatrakul P, Putranto R-A, Martin F, Rio M, Dessailly F, Leclercq J, Dufayard J-F, Lardet L, Montoro P (2012). Some ethylene biosynthesis and AP2/ERF genes reveal a specific pattern of expression during somatic embryogenesis in Hevea brasiliensis. BMC Plant Biol.

[38] Kuswanhadi, Leclercq J, Rio M, Tregear J, Ducamp-Collin M-N, Montoro P (2010). Isolation of three members of the multigene family encoding ACC oxidases in Hevea brasiliensis and Investigation of their responses to ethylene stimulation and wounding. J Rubber Res.

[39] Piyatrakul P, Yang M, Putranto R-A, Pirrello J, Dessailly F, Hu S, Summo M, Theeravatanasuk K, Leclercq J, Montoro P (2014). Sequence and expression analyses of ethylene response factors highly expressed in latex cells from Hevea brasiliensis. PLoS One.

[40] Peng S-Q, Xu J, Li H-L, Tian W-M (2009). Cloning and molecular characterization of HbCOI1 from Hevea brasiliensis. Biosci Biotechnol Biochem.

[41] Tian WW, Huang WF, Zhao Y (2010). Cloning and characterization of HbJAZ1 from the laticifer cells in rubber tree (Hevea brasiliensis Muell. Arg.). Trees.

[42] Zhao Y, Zhou L-M, Chen Y-Y, Yang S-G, Tian W-M (2011). MYC genes with differential responses to tapping, mechanical wounding, ethrel and methyl jasmonate in laticifers of rubber tree (Hevea brasiliensis Muell. Arg.). J Plant Physiol.

[43] Chow K-S, Mat-Isa M-N, Bahari A, Ghazali A-K, Alias H, Mohd-Zainuddin Z, Hoh C-C, Wan K-L (2012). Metabolic routes affecting rubber biosynthesis in Hevea brasiliensis latex. J Exp Bot.

[44] Chow K-S, Wan K-L, Isa M, Bahari A, Tan S-H, Harikrishna K, Yeang H-Y (2007). Insights into rubber biosynthesis from transcriptome analysis of Hevea brasiliensis latex. J Exp Bot.

[45] Li D, Deng Z, Chen C, Xia Z, Wu M, He P, Chen S (2010). Identification and characterization of genes associated with tapping panel dryness from Hevea brasiliensis latex using suppression subtractive hybridization. BMC Plant Biol.

[46] Xia Z, Xu H, Zhai J, Li D, Luo H, He C, Huang X (2011). RNA-Seq analysis and de novo transcriptome assembly of Hevea brasiliensis. Plant Mol Biol.

[47] Li D, Deng Z, Qin B, Liu X, Men Z (2012). De novo assembly and characterization of bark transcriptome using Illumina sequencing and development of EST-SSR markers in rubber tree (Hevea brasiliensis Muell. Arg.). BMC Genomics.

[48] Triwitayakorn K, Chatkulkawin P, Kanjanawattanawong S, Sraphet S, Yoocha T, Sangsrakru D, Chanprasert J, Ngamphiw C, Jomchai N, Therawattanasuk K, Tangphatsornruang S (2011). Transcriptome sequencing of Hevea brasiliensis for development of microsatellite markers and construction of a genetic linkage map. DNA Res.

[49] Liu Q, Kasuga M, Sakuma Y, Abe H, Miura S, Yamaguchi-Shinozaki K, Shinozaki K (1998). Two transcription factors, DREB1 and DREB2, with an EREBP/AP2 DNA binding domain separate two cellular signal transduction pathways in drought- and low-temperature-responsive gene expression, respectively, in Arabidopsis. Plant Cell.

[50] Lorenzo O, Piqueras R, Sánchez-Serrano J, Solano R (2003). ETHYLENE RESPONSE FACTOR1 integrates signals from ethylene and jasmonate pathways in plant defense. Plant Cell.

[51] Sambrook J, Fritsch EF, Maniatis TA (1989). Molecular Cloning: a Laboratory Manual.

[52] Putranto R-A, Sanier C, Leclercq J, Duan C, Rio M, Jourdan C, Thaler P, Sabau X, Argout X, Montoro P (2012). Differential gene expression in different types of Hevea brasiliensis roots. Plant Sci.

[53] Team RC: **R: A language and environment for statistical computing.** In *R Foundation for Statistical Computing.* Edited by Computing RFfS. Vienna, Austria: ᅟ; 2012.

[54] Warnes GR, Bolker B, Bonebakker L, Gentleman R, Liaw WHA, Lumley T, Maechler M, Magnusson A, Moeller S, Schwartz M, Venables B: **gplots: Various R programming tools for plotting data.** In *ᅟ.* 2110th edition; 2012.

[55] Talavera G, Castresana J (2007). Improvement of phylogenies after removing divergent and ambiguously aligned blocks from protein sequence alignments. Syst Biol.

[56] Guindon S, Dufayard J-F, Lefort V, Anisimova M, Hordijk W, Gascuel O (2010). New algorithms and methods to estimate maximum-likelihood phylogenies: assessing the performance of PhyML 3.0. Syst Biol.

[57] Gascuel O (1997). BIONJ: an improved version of the NJ algorithm based on a simple model of sequence data. Mol Biol Evol.

[58] Dufayard J-F, Duret L, Penel S, Gouy M, Rechenmann F, Perrière G (2005). Tree pattern matching in phylogenetic trees: automatic search for orthologs or paralogs in homologous gene sequence databases. Bioinformatics.

[59] Han M, Zmasek C (2009). phyloXML: XML for evolutionary biology and comparative genomics. BMC Bioinformatics.

[60] Chaabouni S, Jones B, Delalande C, Wang H, Li Z, Mila I, Frasse P, Latche A, Pech JC, Bouzayen M (2009). Sl-IAA3, a tomato Aux/IAA at the crossroads of auxin and ethylene signalling involved in differential growth. J Exp Bot.

[61] Curtis M, Grossniklaus U (2003). A gateway cloning vector set for high-throughput functional analysis of genes in planta. Plant Physiol.

[62] Terol J, Domingo C, Talón M (2006). The GH3 family in plants: genome wide analysis in rice and evolutionary history based on EST analysis. Gene.

[63] Chang K, Xiang H, Dunaway-Mariano D (1997). Acyl-adenylate motif of the acyl-adenylate/thioester-forming enzyme superfamily: a site-directed mutagenesis study with the Pseudomonas sp. strain CBS3 4-chlorobenzoate:coenzyme A ligase. Biochemistry.

[64] Paul ES (2008). JAZing up jasmonate signaling. Trends Plant Sci.

[65] Toda Y, Tanaka M, Ogawa D, Kurata K, Kurotani K, Habu Y, Ando T, Sugimoto K, Mitsuda N, Katoh E, Abe K, Miyao A, Hirochika H, Hattori T, Takeda S (2013). RICE SALT SENSITIVE3 forms a ternary complex with JAZ and class-C bHLH factors and regulates jasmonate-induced gene expression and root cell elongation. Plant Cell.

[66] Kazan K (2003). Alternative splicing and proteome diversity in plants: the tip of the iceberg has just emerged. Trends Plant Sci.

[67] Carvalho RF, Feijão CV, Duque P (2013). On the physiological significance of alternative splicing events in higher plants. Protoplasma.

[68] McGlincy N, Smith C (2008). Alternative splicing resulting in nonsense-mediated mRNA decay: what is the meaning of nonsense?. Trends Biochem Sci.

[69] Nicholson P, Mühlemann O (2010). Cutting the nonsense: the degradation of PTC-containing mRNAs. Biochem Soc Trans.

[70] Seo P, Hong S-Y, Kim S-G, Park C-M (2011). Competitive inhibition of transcription factors by small interfering peptides. Trends Plant Sci.

[71] Marquez Y, Brown J, Simpson C, Barta A, Kalyna M (2012). Transcriptome survey reveals increased complexity of the alternative splicing landscape in Arabidopsis. Genome Res.

[72] Heil M, Greiner S, Meimberg H, Krüger R, Noyer J-L, Heubl G, Linsenmair K, Boland W (2004). Evolutionary change from induced to constitutive expression of an indirect plant resistance. Nature.

