# Supplementary material for: Transcriptional and post-transcriptional regulation of the jasmonate signalling pathway in response to abiotic and harvesting stress in Hevea brasiliensis
Source: BMC Plant Biol. 2014 Dec 2;14:341. doi: 10.1186/s12870-014-0341-0 (PMC4274682; doi:10.1186/s12870-014-0341-0)
Supplement: Additional file 5: — Amino acids sequence alignment of HbJAR1 with AtJAR1. [file 12870_2014_341_MOESM5_ESM.docx]

1 10 20 30 40 50 60

| | | | | | |

AtJAR1 ----------------------------------------------------MLEKVETF

HbJAR_20347 ------------------------------------------------------------

HbJAR_21367 ------------------------------------------------------------

HbJAR_20244 ------------------------------------------------------------

HbJAR_5108 ----MAVDYLSSPLGPPACEKDAKALPFIEEMTSNVDSVQERVLAEILSRNSEAEYLKRF

HbJAR_14894 -----------MLERKMEKLNVDKVIEEFEAMTKDTERVQIETLKKILEENGSAEYLQNL

HbJAR_59958 QSSIAVKKFNEDVRKEDGKINIDKVIEEFEAITKDAETIQIETLKKILEENGCAEYLQNL

**I**

AtJAR1 DMNRVTDV-------------ELEPYIKRMVDGDTSPILTGHPVPAISLSSGTSQGRPKF

HbJAR_20347 -------------------------------------------------------MKPKL

HbJAR_21367 ------------------------------------------------------------

HbJAR_20244 ------------------------------------------------------------

HbJAR_5108 PLLGATERDTFKVKIPVVTYEDIQPDIQRIANGDRSAIFCAHPVSEFLTSSGTSAGERKL

HbJAR_14894 GLNGRTDPQSFRDCVPIITHKDMEPYIQRIADGDRSPVLTRKPITTISLSSGTTQGKPKY

HbJAR_59958 GLNGRTDPESFRDYVPIVTHKELEPYIQRIADGDSSSVLTRKPITTISLSSGTTQGKPKY

AtJAR1 IPFTDELMENT---LQLFRTAFAFRNRDFPIDDNGKALQFIFSSKQYISTGGVPVGTATT

HbJAR_20347 IPYFDSALSKAASYLAHNGSNAIFRRLIPPRPVVNKILWFLYADDFTTTRGGFNAVAAST

HbJAR_21367 ------------------------------------------------------------

HbJAR_20244 ------------------------------------------------------------

HbJAR_5108 MPTIYEDMDRR---QLLYSLLMPVMNLYVPGLDKGKGLYFLFVKAETKTPGGLLARPVLT

HbJAR_14894 LPFNDELMEDT---LQIYRTSFAFRNREFPVGN-GKALQFNFSSKQSKTKGGLAAGTATT

HbJAR_59958 LPFNDDLMENT---LQIYRTSFAFRNREFPTVD-GKALLFNFSSKQSRTKGGLAAGTATT

AtJAR1 NVYRNPNFKAGMKSITSPSCSPDEVIFSPDVHQALYCHLLSGILFRDQVQYVFAVFAHGL

HbJAR_20347 --YPLLKMSKAKWSEALSCTSPPEVISGTNVKNQMYCHLLCALRNSDLIDGIRAPYAIGL

HbJAR_21367 ------------------------------------------------------------

HbJAR_20244 ------------------------------------------------------------

HbJAR_5108 SYYKSEQFKNRPYDPYNVYTSPNETILCADSFQSMYAQMLCGLIMREEVLRVGAVFASGL

HbJAR_14894 NLFRNSHFKNAVQTMQLKCCSPDEVVFGSDFHQSLYCHLLCGLIFREEIQFVSSTFAHSI

HbJAR_59958 NLFRSSCYKNAVRTMQFICCSPDEVIFGSDFHQSLYCHLLCGLIVREEIQFVFSTFAHSI

AtJAR1 VHAFRTFEQVWEEIVTDIKDGVLSNRITVPSVRTAMSKLLT-PNPELAETIRTKCMSLSN

HbJAR_20347 VKAFNLLESKWGQLCDDLENGFPDMQITDPAMRESVAKVLNGPQPDLSNRLRS-IFEEKN

HbJAR_21367 ------------------------------------------------------------

HbJAR_20244 ------------------------------------------------------------

HbJAR_5108 LRAIKFLQINWKQLVEDISSGTLNPKVTDHSVRECMTKILK-PNPDLAEFITKQCSGE-N

HbJAR_14894 VLAFRTFEQVWEELCDNIRDGVLSSRVTDSSIRNAISKLLK-PNFELAELIHEKCLGLSN

HbJAR_59958 VLAFRTFEQVWEELCDNIRDGMLSSRVTDPSIRMQ-------------------------

**II**

AtJAR1 WYGLIPALFPNAKYVYGIMTGSMEPYVPKLRHYAGDLPLVSHDYGSSEGWIAANVTPRLS

HbJAR_20347 WGGIVSKLWSNVRYVKCVTTGSMKHYYSKLKYYAGEVIILGGDYFASECPVGINLDTKQP

HbJAR_21367 ------------------------------------------------------------

HbJAR_20244 --------------------------------------MASTMYASSECYFGLNLKPICK

HbJAR_5108 WEGIITRIWPNTKYLDVIVTGAMAQYIPTLEYYSAGLPMACTMYASSECYFGLNLKPMSK

HbJAR_14894 WYGLIPELFPNVKYVYGIMTGSMEPYLKKLRHYSAEIPLLSGDYGATEGWIAANVNPKLP

HbJAR_59958 ------------------------------------------------------------

AtJAR1 PEEATFAVIPNLGYFEFLPVS-----ETGEGEEKPVGLTQVKIGEEYEVVITNYAGLYRY

HbJAR_20347 PEATRFAMLPTAAYFEFLPFDLN---EGSVVGKETVNFSGVEVGKTYEVVVTTYRGIYRY

HbJAR_21367 -----------MAYFEFLPHEPHSAGLTCDSPPKLVDLVDVELGKEYELVITTYAGLCRY

HbJAR_20244 PSEVCYTIMPNMAYFEFLPREPS--APSRDCPPRLLDLADVEVGKEYELVITTYSGLYRY

HbJAR_5108 PSEVSYTIMPNMAYFEFLPHEPSAPAPSRDCPPRLVDLANVEVGKEYELVITTYSGLYRY

HbJAR_14894 PELVTFAVLPNIGYFEFISLGDN--VDKIYTEPKPVGLTEVKIGEEYEIIVTNFAGLYRY

HbJAR_59958 ------------------------------------------------------------

**III**

AtJAR1 RLGDVVKVIGFYNNTPQLKFICRRNLILSINIDKNTERDLQLSVESAAKRLSE-EKIEVI

HbJAR_20347 RLGDIVRVVGFHNSSPEVEFVMRAP---KNAYEVITERDLMSAVESFQLVMRNAIAAEIV

HbJAR_21367 RVGDILRVTGFHNSAPQFHFVRRKNVLLSIDSDKTDEAELQKAVENASQLLRE-YNTSVV

HbJAR_20244 RVGDILLVTGFYNNAPQFRFLRRKNVLLSIDVDKTDEAELQKGIENASLLLRE-FNTSVV

HbJAR_5108 RVGDILRVTGFYNKAPQFRFVRRKNVLLSIDSDKTDEAELQKGIENASALLRE-FNTSVV

HbJAR_14894 RLGDVVKVMGFHNSTPELKFVCRRSLLLTINIDKNTEKDLQLSVEEAAKLLAG-EKLELV

HbJAR_59958 ------------------------------------------------------------

AtJAR1 DFSSYIDVSTDPGHYAIFWEI-----SGETNEDVLQDCCNCLDRAFIDAGYVSSRKCKTI

HbJAR_20347 EF-----------------------------------AR??LEEIL--------------

HbJAR_21367 EYTSYADTETIPGHYVIYWELLIKDSTNSPSEKVLSECCVAMEESMNSVYRQGRVADNSI

HbJAR_20244 EYTSYAETKTIPGHYVIYWELLIKDPSSSPTVEVLNQCCLAIEESLNMVYRRGRVADNSI

HbJAR_5108 EYTSYADTKSIPGHYVIYWELFVKDPANSPTEEVLNQCCLAMEESLNSVYRQGRVADNSI

HbJAR_14894 DFSSLVDLSTEPGHYVIFWEI-----SGEPTEEVLKECCNCMDRSFLDAGYITSRKINAI

HbJAR_59958 -------------------------------------CHKCLSQT*--------------

AtJAR1 GALELRVVAKGTFRKIQEHFLGLGSSAGQFKMPRCVKPSNAKVLQILCENVVSSYFSTAF

HbJAR_20347 --------------------------------------SRNAHVELTCKRAWP*------

HbJAR_21367 GPLEIRVVKNGTFEELMDYAISRGASINQYKVPRCV--NFTPIMELLDSRVVSRHFSPSL

HbJAR_20244 GPLEIRVVKNGTFEELMDYAISRGASINQYKVPRCV--SFTPIMELLDSRVVSNHFSPSL

HbJAR_5108 GPLEIRVVKNGTFEELMDYAISRGASINQYKVPRCV--SFTPITELLDSRVVSKHFSPSL

HbJAR_14894 GPLELRVVRKGTFQKILDHYLGLGAAVSQFKTPRCIGPTNNVVLQILSNNVAKTYHSCAF

HbJAR_59958 ------------------------------------------------------------

AtJAR1 -----------

HbJAR_20347 -----------

HbJAR_21367 PHWTPERRRL*

HbJAR_20244 PHWTPERRR*-

HbJAR_5108 PHWTPERRR*-

HbJAR_14894 *----------

HbJAR_59958 -----------
