# Supplementary material for: Transcriptional and post-transcriptional regulation of the jasmonate signalling pathway in response to abiotic and harvesting stress in Hevea brasiliensis
Source: BMC Plant Biol. 2014 Dec 2;14:341. doi: 10.1186/s12870-014-0341-0 (PMC4274682; doi:10.1186/s12870-014-0341-0)
Supplement: Additional file 6: — Amino acids sequence alignment of HbCOI with AtCOI. [file 12870_2014_341_MOESM6_ESM.docx]

1 10 20 30 40 50 60

| | | | | | |

AtCOI_At2g39940 --MEDPDIKRCKLSCVATVDD-VIEQVMTYITDPKDRDSASLVCRRWFKIDSETREHVTM

HbCOI_2304 MEEENQSNKSSRISCSSGMSDVVLGCVMPYIHDPRDRDAVSLVCRRWYELDALTRKHITI

HbCOI_3058 ---------------MNYFPDEVLEHVFDFVTSHRDRNSLSQVCKSWYRIESSSRQKVFI

F-BOX

AtCOI_At2g39940 ALCYTATPDRLSRRFPNLRSLKLKGKPRAAMFNLIPENWGGYVTPWVTEISNNLRQLKSV

HbCOI_2304 ALCYTTSPDRLRRRFKHLESLKLKGKPRAAMFNLIPEDWGGFVTPWVNEIAESFNCLKSL

HbCOI_3058 GNCYAISPERVIERFPGLKSITLKGKPHFADFNLVPNDWGGFVYPWIEAFARNRVGLEEL

AtCOI_At2g39940 HFRRMIVSDLDLDRLAKARADDLETLKLDKCSGFTTDGLLSIVTHCRKIKTLLMEESSFS

HbCOI_2304 HFRRMIVKDSDLELLAKSRGRVLQVLKIEKCSGFSTDGLLHVGRLCRQLRTLFLEESSIL

HbCOI_3058 RLKRMVVSDESLELLSRCFPN-FKSLVLVSCEGFTTDGLAAIAANCRFLRELDLQENEVE

LRR

AtCOI_At2g39940 EKDGKWLHELAQHNTSLEVLNFYMTEFAKISPKDLETIARNCRSLVSVKVGDFEILELVG

HbCOI_2304 EKDGDWLHEIALNNTVLETLNFYMTDLNTVRFEDLELIAKNCRNLVSVKISDCEILDLVG

HbCOI_3058 DHRGHWLGCFPDSCTSLTSLNFACLK-GDINLGVLERLVARSPNLRSLRLNRAVPLDTLQ

AtCOI_At2g39940 --FFKAAANLEEFCGGSLNEDIGMPEKYMNLVFPRKLCR-----LGLSYMGPNEMPILFP

HbCOI_2304 --FFHAAAALEEFCGGSFNDV---PERYSAVSFPRKLCR-----LGLTYMGKNEMPIVFP

HbCOI_3058 KILMQSPQLLDLGVGSYVNDPD--SEIYSKLVTAIQKCESVRSLSGFLDVAPHCLPAFHS

AtCOI_At2g39940 FAAQIRKLDLLYAL-LETEDHCTLIQKCPNLEVLETRNVIGDRGLEVLAQYCKQLKRLRI

HbCOI_2304 FASLLKKLDLLYAL-LDTEDHCLLIQKCCNLEVLETRNVIGDRGLEVLASSCKRLKRLRI

HbCOI_3058 ICPNLTSLNLSYAPGIHGSELTKLIHHCRKLQRLWILDCIGDKGLEVVASTCKDLLELRV

AtCOI_At2g39940 ERGADEQGMEDEEGLVSQRGLIALAQGCQELEYMAVYVSDITNESLESIGTYLKNLCDFR

HbCOI_2304 ERGADEQGMEDEEGVVSQRGLIALAQGCLELEYMAVYVSDITNAALEHIGTHLRKLNDFR

HbCOI_3058 FPSDPYVG----NAAVTEEGLVAISSGCPKLNSILYFCQQMTNAALITVAKNCPNFTRFR

LRR

AtCOI_At2g39940 LVLLD--REERITDLPLDNGVRSLLIGCKKLRRFAFYLRQGGLTDLGLSYIGQYSPNVRW

HbCOI_2304 LVLLD--REERITDLPLDRGVQSLLMQ-RKLRRFALYLRPGGLTDEGLGYIGQHSKNVRW

HbCOI_3058 LCILDPTKPDAVTMQPLDEGFGAIVHSCRGLRRLSL---SGLLTDQVFLYIGMYAEQLEM

LRR

AtCOI_At2g39940 MLLGYVGESDEGLMEFSRGCPNLQKLEMRGCCFSERAIAAAVTKLPSLRYLWVQGYRASM

HbCOI_2304 MLLGYVGESDEGLLAFSKGCPSLQKLEMRGCCFTEGALAKAVMQLTSLRYLWVQGYRASS

HbCOI_3058 LSIAFAGDSDKGMQYVLNGCKKLRKLEIMDCPFGNGALLMDVGKYETMRSLWMSSCEVTL

LRR

AtCOI_At2g39940 T-GQDLMQMARPYWNIELIPSRRVPEVNQQGEIREMEHPAHILAYYSLAGQRTDCPTTVR

HbCOI_2304 TRGRDLLAMARPFWNIELIPPRKVVMVNQVGEDVVVEQPAHILAYYSLAGPRTDFPNTVV

HbCOI_3058 G-GCKTLAKMMPRLNVEIMNENELADFSADHTPKVNK----MYLYRTLVGRRKDAPEYVW

AtCOI_At2g39940 VLKEPI------

HbCOI_2304 PLDSCRIESCK*

HbCOI_3058 TL*---------
