# Supplementary material for: Transcriptional and post-transcriptional regulation of the jasmonate signalling pathway in response to abiotic and harvesting stress in Hevea brasiliensis
Source: BMC Plant Biol. 2014 Dec 2;14:341. doi: 10.1186/s12870-014-0341-0 (PMC4274682; doi:10.1186/s12870-014-0341-0)
Supplement: Additional file 7: — Amino acids sequence alignment of HbJAZ with AtJAZ1. [file 12870_2014_341_MOESM7_ESM.docx]

1 10 20 30 40 50 60

| | | | | | |

AtJAZ1_At1g19180 ---------------------------------------------------MSSSMECSE

HbJAZ_14313 ------------------------------------------------------------

HbJAZ_1229 ---------------------------------------------------MEGESDSYR

HbJAZ_863 ---------------------------------------------------MEGDSDSYE

HbJAZ_19967 ----------------------------------------------MRKIILSDMANLVQ

HbJAZ_1660 ---------------------------------------------------MAGSPEFVE

HbJAZ_26925 ------------------------------------------------------------

HbJAZ_29511 -----------------------------------------MCIFVSILTILKNFLLIFL

HbJAZ_1405 ------------------------------------------------------------

HbJAZ_17062 MMERDF--WVWVESVRTVKEEVT-DGYKDSVPMRGSAMQWSFLKKVSAIPQFLSFKSGEE

HbJAZ_2001 -MERDFMGLNSKEPLAVVKEEVNCDGYKEIGFSKSSGIHWPFSNKVSALPHLNSFKVSQE

AtJAZ1_At1g19180 FVGSRRF-TGKKPSFSQTCSRLSQYLKENGSFGDLSLGMACKPDVNGTLG----------

HbJAZ_14313 SLFRRNHRNPLSLSKFRNDEHTDHLEEEEEDIVGGGEESVENPNIHYENG----------

HbJAZ_1229 EVKPKAGEDQLVPVNKSSLAATNGYDENLGSCKQGVLLQTNSS-----------------

HbJAZ_863 EVKAKAGEE---------LPATNGADENMGSCKEGVLPWTNSS-----------------

HbJAZ_19967 NKSSGKA-APEKSNFAQTCNLLSQYLKERGSFRDLSLGINGKLEAKGPEA----------

HbJAZ_1660 FGGRKAAKSAEKSSFSQTCSLLSQYIKEKGSFGDLSLGMTCSAEGNGN-G----------

HbJAZ_26925 ------------------------------------------------------------

HbJAZ_29511 QIFRALYQKSILSFSSLSLPLVLPISRVLQKWLSLRFQKSFSV-----------------

HbJAZ_1405 ------------------------------------MRRNCNL-----------------

HbJAZ_17062 ESPRKTIHDPIASSGFTPISTADALDSNQKQYSSM-IQKNMALDRQGANHYAMTAYAVQH

HbJAZ_2001 DKTKRLVSDSSLSPGFLSISTADAFDSNQKQFMAE-IQKSFNHNRQSGTDFTLTAYPVQH

AtJAZ1_At1g19180 ---NSRQPTTTMSLFPCEASNM----------------DSMVQDVKPTNLFPRQPSFSSS

HbJAZ_14313 ---------------------------------------------------NGTDGGVDG

HbJAZ_1229 ------------------------------------------------------------

HbJAZ_863 ------------------------------------------------------------

HbJAZ_19967 -S---RPPTTTLNLLSNIEISA--------EISRQNS--VLSANIKPMDFFPQFVGFASP

HbJAZ_1660 -TTELRQAATTMNLFPMSEKQV--------DVSSRNMATPPRTNFRSMDLFPQQAGFSPS

HbJAZ_26925 ------------------------------------------------------------

HbJAZ_29511 -----PAAPKEQTPFP--------------------------------------------

HbJAZ_1405 ----------ELQLFPFSDPDH--------------------------------------

HbJAZ_17062 VDAYPVHRPQQMRIFPVINHQNPTITVSMSNPNLQSHFASTGNNVGGKSINSQYLAGVPI

HbJAZ_2001 -DVHSVHHPRDMKMFPVSNHAS---SISLSNPFFKNYYAPSGQNVSGATVKPQLLGGIPV

TIFY

AtJAZ1_At1g19180 SSSLP-KEDVLKMTQTTR---SVKPESQTAPLTIFYAGQVIVFNDFSAEKAKEVINLASK

HbJAZ_14313 V-----LPSGIRDTGGPDYPLVVANGGNADQLTLSFHWEVYVFDAVAPDKVQAVLLLLGG

HbJAZ_1229 ------RNASMPTSGTNA------TIPTSDQLTIFYGGSVLVFDAIPAETAREIMLIAAA

HbJAZ_863 ------RPATMATSGPNA------TVPTSDQLTIFYGGSILVFDTIPAEKVREIMLIAAA

HbJAZ_19967 N---PIEEDSIANKPADL-RKSSREEPGTAQLTIFYAGQVIVYDDFPADKAKEIMALASK

HbJAZ_1660 A---P-KEDVQKSLDSSV-NKAATPEPQTAPMTIFYAGQVIVFNDFPADKVKEVMLLASK

HbJAZ_26925 -------------------------------MTIFYCGKVNVYDGIPPDKAQAIMHLAAS

HbJAZ_29511 ------RLPVYYPLQRPA----LENPPQTAPLTIFYNGTVAVF-DVPRDTAESILKLAEN

HbJAZ_1405 ------HPPVLYEKETSD----EQSPQHSQQLTIFYNGRVCVC-DVTELQARAILLLASR

HbJAZ_17062 VSPVSVHPTPSSVVGTTDLRNRSKSSGAPAQLTIFYAGSVCVYEDISPEKAQAIMLLAGH

HbJAZ_2001 TTPQTILPTVGSVTGMME--SCAIASGSPAQLTIFYGGTVNVYDDISPEKVQAIMFLAGQ

AtJAZ1_At1g19180 -GTANSLAKNQTDIRSN--------------------------IATIANQVPHP------

HbJAZ_14313 CEIPSSIPTAGTVPLNLTVPSDLPGRSIQPQRAASLHRFREKRKELCFDKKIRYSVRKEV

HbJAZ_1229 -AAAAVKPADMKKAVSGSPAG----------------------GTPVLTRSPSL------

HbJAZ_863 -AAVAVKPADMKKAISGSPAG----------------------GTPVLTRSPSP------

HbJAZ_19967 -GTSNSKNGFTTTA-----------------------------STSAMDKTNSI------

HbJAZ_1660 -GSSQSLTGFPSVPVKSHPVFDPNVA-----------------KAPVESTSSIP------

HbJAZ_26925 --------------RIQSPMDDPI-------------------RRPAFSFPCHF------

HbJAZ_29511 -GF---------------------------------------------------------

HbJAZ_1405 -EMEENL---------RTPVGTP-------------------------------------

HbJAZ_17062 -GSSVTQNKAISPVQVRTPIPRPLTPIPRPSADDGFVGSKIHTASPCSGLPSPI------

HbJAZ_2001 -DSSISSNMALPKIQVHAPSSKPIATDVNPVNHN-------VTTPPCSRLSSPL------

AtJAZ1_At1g19180 -----------------------------------------RKTTTQEPIQSSPTPLTEL

HbJAZ_14313 ALRMQRKKGQFASSKASSDEAGSASSGWSATQGSGQDDMLETSCNHCGTSSKSTPLMRRG

HbJAZ_1229 ------------------------------QSTTSALASPQTQLYSVHQGSSLCKLQAEL

HbJAZ_863 ------------------------------QSSTSALPSPQAQVLPVHQGFSLRKLQAEL

HbJAZ_19967 ---------------------------------------ASNNNAREGLRLQTQANGSDL

HbJAZ_1660 -------------------------------PNSNPVPSFGNNLNQERVQSPSQTIASDL

HbJAZ_26925 ----------------------------------QTQSDKHGFIPPNAAISLANQTDVEG

HbJAZ_29511 ----------------------------------------SKAVESTNQKQVLESLEGDL

HbJAZ_1405 ---------------------------------------TGSEVASPSLPSPLCTPMAAG

HbJAZ_17062 --SVT----------SSSAIELTTVKSVGALASANNPIESSKTVSSAAPGSAIPIPAGAV

HbJAZ_2001 --SVSSQTGAQSGSGSTSTEEIMATKTTGVATTPVSKLDTPKLTSAMGSVAATTMMPSAV

AtJAZ1_At1g19180 PIARRASLHRFLEKRKDR------------------------------------VT--SK

Jas

HbJAZ_14313 PAGPRTLCNACGLKWANKGILRD-------------------------------LSKISR

HbJAZ_1229 PIARRHSLQRFFEKRRDR------------------------------------LC--SK

HbJAZ_863 PITRRHSLQRFFEKRRDR------------------------------------LC--SK

HbJAZ_19967 PIARRASLHRFFEKRKDR------------------------------------VA--SK

HbJAZ_1660 PIARRASLHRFLEKRKDR------------------------------------ITASAR

HbJAZ_26925 QANRKVLLQRYLEKKKDRGRFKGRKNTGPTSSSLEVYLNHHGKMQSANGQSTRSST--SS

HbJAZ_29511 PIARRKSLQRFLEKRKER------------------------------------LT--SL

HbJAZ_1405 -LSMKRSIQRFLQKRKHR------------------------------------VQ--AI

HbJAZ_17062 PQARKASLARFLEKRKER------------------------------------VM--NT

HbJAZ_2001 PQARKASLARFLEKRKER------------------------------------VM--SA

AtJAZ1_At1g19180 APYQLCDPAKASSNPQTTGNMSW---LGLAAEI-----------------

HbJAZ_14313 MAIQGPPAKSIEQSEGKANGMDAVTVAADIVSSFNGDNSTLTAET*----

HbJAZ_1229 SPYPTPQAMKMAETTKPDFSAEVSPEAGCFGKTLAPEKEIQPKVTANLA*

HbJAZ_863 SPYPSPPAMKMAETIKPEFSAQVSPDAGCFGKPLAPEREIQPKVAANLA*

HbJAZ_19967 APYQLNNPSSPARPRPYEESNPIIIDLEVEGQSSKQLELKL*--------

HbJAZ_1660 APYQTS-RGLSASPSKPAESKPW---LGLAGQSLQ*--------------

HbJAZ_26925 PPQPGVPPTLCSSAEDQSKIAGFSVDLNEDVQDC*---------------

HbJAZ_29511 SPYACTPDCRS*--------------------------------------

HbJAZ_1405 SPYNH*--------------------------------------------

HbJAZ_17062 SPYNVSKKSLDCSAAECDDVSLSINSLSSPGHQ*----------------

HbJAZ_2001 APYNMGKKSPESAMQNPIE*------------------------------
