# Supplementary material for: Transcriptional and post-transcriptional regulation of the jasmonate signalling pathway in response to abiotic and harvesting stress in Hevea brasiliensis
Source: BMC Plant Biol. 2014 Dec 2;14:341. doi: 10.1186/s12870-014-0341-0 (PMC4274682; doi:10.1186/s12870-014-0341-0)
Supplement: Additional file 8: — Amino acids sequence alignment of HbMYC with AtMYC2. [file 12870_2014_341_MOESM8_ESM.docx]

1 10 20 30 40 50 60

| | | | | | |

AtMYC2_At1g32640 MTDYRLQPTMNLWTTDDNASMMEAFMSSSDISTLWPPASTTTTTATTETTPTPAMEIPAQ

HbMYC_424 -------------------------MQKISMDELTSPSSSSSFMSFCQDSYPP-------

HbMYC_771 -------------------------MKKISMEEIASPSSSSSFMSFCQDSSPP-------

HbMYC_94937 ------------------------------MEEITSPSSTSSFMSFCQDTCPP-------

II

I

AtMYC2_At1g32640 AGFNQETLQQRLQALIEGTHEGWTYAIFWQPSYDFSGASVLGWGDGYYKGEED-KANPRR

HbMYC_424 -------LQQRLQFILQSRPEWWVYAIFWQASKDATGRLVLSWGDGHFRGTEEFAAKACC

HbMYC_771 -------LQQRLQFILQSRPEWWVYAIFWQASKDATGRLVLSWGDGHFRGTKEFAAKACN

HbMYC_94937 -------LQQRLQFILQSRPEWWVYAIFWQASKDATGRLVLSWGDGHFRGTKEFAAKACN

III

AtMYC2_At1g32640 RSSSPPFSTPADQEYRKKVLRELNSLISGGVAPSDDAVDEEVTDTEWFFLVSMTQSFACG

HbMYC_424 KQNQLKFGFNLE---RKMTNKESQTLFSDDME-MDRLADVDAIDYEWFYTVSVTRSFAVE

HbMYC_771 KQNQPKFGFNLE---RKMINKESQTLFTDDMD-MDRLADVDVIDYEWFYTVSVTRSFAID

HbMYC_94937 KLNQPKFGFNLE---RKMINKESPTIFGDDMD-MDRLADVEVIDYEWFYTVSVTRSFAVE

JAZ interacting domain

IV

AtMYC2_At1g32640 AGLAGKAFATGNAVWVSGSDQLSGSGCERAKQGGVFGMHTIACIPSANGVVEVGSTEPIR

HbMYC_424 DGILGKTFGSWAFIWLTGNHELQMYECERVKEARMHGVQTLVCISTTCGVVELGSSNTID

HbMYC_771 DGILGRTFGSGAFIWLTGNNELQMYDCERVKEARMHGIQTLVCISTSCAVVELGSSNTID

HbMYC_94937 DGILGRAFGSGAFIWLTGNHELQMFGCERVKEARMHGIQTLACISTTCGVVELGSSNTID

AtMYC2_At1g32640 QSSDLINKVRILFNFDGGAGDLSGLNWNLDPDQGENDPSMWINDPIGTPGSNEPGNGAPS

HbMYC_424 KDWSLVQLCKSI------------------------------------------------

HbMYC_771 KDWSLVQLCKSL------------------------------------------------

HbMYC_94937 KDWSLVQLCKSL------------------------------------------------

AtMYC2_At1g32640 SSSQLFSKSIQFENGSSSTITENPNLDPTPSPVHSQTQNPKFNNTFSRELNFSTSSSTLV

HbMYC_424 -----------FGGDTACLVSKEPSHE-------SQIQIP--NTCLLDIGTFSASQKETF

HbMYC_771 -----------FGGDTACLVSKEPSHE-------SQLQIP--NTSFLDIGMFSASQKDTS

HbMYC_94937 -----------FEGDSACLVSKEPSHD-------SQLHIL--NTSFLDISMFSASQKETS

AtMYC2_At1g32640 KPRSGEILNFGDE-GKRSSGNPDPSSYSGQTQFENKRKRSMVLNEDKVLSFGDKTAGESD

HbMYC_424 TQKQNE----DDKNKKDATGQGRSSSDSARSDSD-----------------GNFTAGNTD

HbMYC_771 AEKQNE----GDK-KKDPTGQGRSSSDSARSDSE-----------------GNFAAGNTD

HbMYC_94937 TEKQIE----GDK-KKDVTGQGRSSSDSARSDSD-----------------GNFAAGNTD

bHLH

AtMYC2_At1g32640 HSDLEASVVKEVAVEKRPKKRGRKPANGREEPLNHVEAERQRREKLNQRFYALRAVVPNV

HbMYC_424 ----------------RFKKRGRKQLNGEELPINHVEAERQRRERLNHRFYALRSAVPNV

HbMYC_771 ----------------RLKKRGRTQLNGKELTLNHVEAERQRRERLNHRFYALRSVVPNV

HbMYC_94937 ----------------RFKKRGRKQLNGKELPLNHVEAERQRRERLNHRFYALRSVVPNV

AtMYC2_At1g32640 SKMDKASLLGDAIAYINELKSKVVKTESEKLQIKNQLEEVKLELAGRKASASGGDMSSSC

HbMYC_424 SKMDKASLLADAVTYIKELKATVDELQS-KLEAVSKKSKST-NVTDNQSTDSMIDHMRSS

HbMYC_771 SKMDKASLLADAVTYIKELKAKVDELES-KLQAVSKKSKIT-SVTDNQSTDSMIDHIRSS

HbMYC_94937 SKMDKASLLADAVTYIKELKAKVDELES-KLQAVSKKSKST-NVADNQSTDSMIDHIRAS

AtMYC2_At1g32640 SSIKPVGMEIEVKIIGWDAMIRVESSKRNHPAARLMSALMDLELEVNHASMSVVNDLMIQ

HbMYC_424 SSYKAKGMELDVTIVGSEAMIRFLSPDVNYPAARLMDVLREVEFKVHHASMSSIKEMVLQ

HbMYC_771 SAYKAKAMELEVKIVGSEAMIRFLSPDVNYPAARLMDALREVEFKVHHASMSSIKEMVLQ

HbMYC_94937 SIYKTKAMELEVKIVGSEAMIRFLSPDVNYPAARLMDVLREIEFKVHHASMSSIKEMVLQ

AtMYC2_At1g32640 QATVKMGFRIYTQEQLRASLISKIG--

HbMYC_424 DVVVRVPDGLTDEEVVRSAILQRMQN*

HbMYC_771 DVVARVPDGLTNEELVRSAILQRMQN*

HbMYC_94937 DVVARVPDGLTNEDVVRSAILQRMQN*
