# Supplementary material for: Transcriptional and post-transcriptional regulation of the jasmonate signalling pathway in response to abiotic and harvesting stress in Hevea brasiliensis
Source: BMC Plant Biol. 2014 Dec 2;14:341. doi: 10.1186/s12870-014-0341-0 (PMC4274682; doi:10.1186/s12870-014-0341-0)
Supplement: Additional file 9: — Amino acids sequence alignment of HbNINJA with AtNINJA. [file 12870_2014_341_MOESM9_ESM.docx]

>Protein alignment 172 Alignment of 2 sequences: AtNINJA_At4g28910, HbNINJA_632
8


Identities = 243/525 (46%), Positives = 305/525 (58%), Gaps = 115/525 (21%)

AtNINJA_At4g28910     1 MDDDNGLELSLGLSCGGSTGKAKGNNNNNAGSSSENYRAEGGDRSAKVIDDFKNFLHPTS  60 
                        M+D+NGLELSLGL CGGS+ K+KG N       S + R E GDR  K++DDFKNFLH ++     
HbNINJA_6328          1 MEDENGLELSLGLGCGGSSAKSKGKN------GSSDTRTEEGDRGNKLVDDFKNFLHAST  54 

AtNINJA_At4g28910    61 QRPAEPSSGSQRSDSGQQPPQNFFNDLSKAPTTEAEAS----TKPLWV---------EDE 107 
                        Q+  + S+GSQ SDS  +P +NFFNDLSK    +A AS     + LWV         E+E     
HbNINJA_6328         55 QKQ-DSSAGSQISDS-VKPQENFFNDLSKG-NADANASINLNNRGLWVSSGKRPAEIEEE 111 

AtNINJA_At4g28910   108 SRKEAGNKRKFGFPGMNDDKKKEKDSSHVDMHEKKTKASHVSTATDEGSTAENEDVAESE 167 
                         R EAGNKRK  F  +N+ KK E+D+ H D+H+K  KASH+S  T++GSTAENEDVAESE     
HbNINJA_6328        112 KRPEAGNKRKMLFDEINNQKKHERDAYHSDVHDK--KASHISITTEDGSTAENEDVAESE 169 

AtNINJA_At4g28910   168 VGG-----------------GSSSNHAKEVVRPPTDTNIVDNLTGQRRSNHGGSGTEEFT 210 
                        V G                 G+      + V   +D+++VD L GQ+R N  GS   E       
HbNINJA_6328        170 VEGSISRLISHHDDGPKRFIGAGGPEVPKEVHGFSDSSVVD-LQGQKRPN--GSSESEIK 226 

AtNINJA_At4g28910   211 MRNMSYTVPFTVHPQNVVTSMPYSLPTKESGQHAAATSLLQP------------------ 252 
                          N++Y VPF+V P N++ ++PYS P KES      +S   P                       
HbNINJA_6328        227 HGNLNYGVPFSVRPVNIM-NLPYSFPVKESNTIGVPSSSGHPLPGMMQVTSNGEQRTGTQ 285 

AtNINJA_At4g28910   253 NANAGNLPIMFGYSPVQLPMLDKDGSGGIVALSQSPF---AGRVPSNSAT---------- 299 
                        + N GNLP+MFGYSPVQLP LDKD S G+V+  Q      AGR PSNS                 
HbNINJA_6328        286 SVNPGNLPVMFGYSPVQLPTLDKDNSWGLVSHLQQLHPSCAGRGPSNSDKQNDVLKIAPA 345 

AtNINJA_At4g28910   300 ---------------------AKGEGKQPVAEEGSSEDASERPTGDNSNLNT-------- 330 
                                              KG+GKQ + EEG +    +   G + NL               
HbNINJA_6328        346 MQGISRNSSEATLYEGRTLERVKGDGKQHITEEGFTSQTEDDVKGSSMNLGAKDAPGPST 405 

AtNINJA_At4g28910   331 --AFSFDFSAIKPGMAADVKFGGSGARPNLPWVSTTGSGPHGRTISGVTYRYNANQIKIV 388 
                           FS+DFSAIKPG+A+D+KFGG G+ PNLPWVSTTGSGPHGRTISGVTYRY+ANQI+IV     
HbNINJA_6328        406 AEGFSYDFSAIKPGIASDIKFGGCGSYPNLPWVSTTGSGPHGRTISGVTYRYSANQIRIV 465 

AtNINJA_At4g28910   389 CACHGSHMSPEEFVRHASEEYVSPESSMGMT-------AASAHT- 425 
                        CACHGSHM PEEFVRHASEE V+P++  G+        AASA +      
HbNINJA_6328        466 CACHGSHMLPEEFVRHASEENVNPDNGTGVASFPSANPAASAQS* 510
