# Supplementary material for: Transcriptional and post-transcriptional regulation of the jasmonate signalling pathway in response to abiotic and harvesting stress in Hevea brasiliensis
Source: BMC Plant Biol. 2014 Dec 2;14:341. doi: 10.1186/s12870-014-0341-0 (PMC4274682; doi:10.1186/s12870-014-0341-0)
Supplement: Additional file 10: — Amino acids sequence alignment of HbTPL with AtTPL. [file 12870_2014_341_MOESM10_ESM.docx]

>Protein alignment 173 Alignment of 2 sequences: AtTPL_At1g15750, HbTPL_7591

Identities = 979/1145 (85%), Positives = 1054/1145 (92%), Gaps = 19/1145 (1%)

AtTPL_At1g15750      1 MSSLSRELVFLILQFLDEEKFKETVHKLEQESGFFFNMKYFEDEVHNGNWDEVEKYLSGF   60 
                       MSSLSRELVFLILQFLDEEKFKETVHKLEQESGFFFNMKYFEDEVH+GNWDEVEKYLSGF      
HbTPL_7591           1 MSSLSRELVFLILQFLDEEKFKETVHKLEQESGFFFNMKYFEDEVHSGNWDEVEKYLSGF   60 

AtTPL_At1g15750     61 TKVDDNRYSMKIFFEIRKQKYLEALDKHDRPKAVDILVKDLKVFSTFNEELFKEITQLLT  120 
                       TKVDDNRYSMKIFFEIRKQKYLEALDKHDR KAVDILVKDLKVF+TFNEELFKEITQLLT      
HbTPL_7591          61 TKVDDNRYSMKIFFEIRKQKYLEALDKHDRSKAVDILVKDLKVFATFNEELFKEITQLLT  120 

AtTPL_At1g15750    121 LENFRENEQLSKYGDTKSARAIMLVELKKLIEANPLFRDKLQFPTLRNSRLRTLINQSLN  180 
                       LENFRENEQLSKYGDTKSARAIMLVELKKLIEANPLFRDKLQFP L+NSRLRTLINQSLN      
HbTPL_7591         121 LENFRENEQLSKYGDTKSARAIMLVELKKLIEANPLFRDKLQFPNLKNSRLRTLINQSLN  180 

AtTPL_At1g15750    181 WQHQLCKNPRPNPDIKTLFVDHSCGPPNGARAPSPVNNPLLGGIPKAGGFPPLGAHGPFQ  240 
                       WQHQLCKNPRPNPDIKTLFVDHSCG PNGARAPSP NNPLLG +PKAGGFPPLGAHGPFQ      
HbTPL_7591         181 WQHQLCKNPRPNPDIKTLFVDHSCGQPNGARAPSPANNPLLGSLPKAGGFPPLGAHGPFQ  240 

AtTPL_At1g15750    241 PTASPVPTPLAGWMSSPSSVPHPAVS-AGAIALGGPSIPAALKHPRTPPTNASLDYPSAD  299 
                       PTA PVP PLAGWMS+PS+V HPAVS  GAI LG PSIPAALKHPRTPPTN S+DYPS D      
HbTPL_7591         241 PTA-PVPAPLAGWMSNPSTVTHPAVSGGGAIGLGAPSIPAALKHPRTPPTNPSVDYPSGD  299 

AtTPL_At1g15750    300 SEHVSKRTRPMGISDEVNLGVNMLPMSFSGQAHGHSPAFKAPDDLPKTVARTLSQGSSPM  359 
                       S+HV+KRTRPMGISDEVNL VN+LP+SF G  HGH   F APDDLPKTVAR+L+QGSSPM      
HbTPL_7591         300 SDHVAKRTRPMGISDEVNLPVNVLPVSFPG--HGHGQTFNAPDDLPKTVARSLNQGSSPM  357 

AtTPL_At1g15750    360 SMDFHPIKQTLLLVGTNVGDIGLWEVGSRERLVQKTFKVWDLSKCSMPLQAALVKEPVVS  419 
                       SMDFHP++QTLLLVGTNVGD+GLWEVGSRERLV + FKVWDLS CSMPLQAAL K+PVVS      
HbTPL_7591         358 SMDFHPVQQTLLLVGTNVGDVGLWEVGSRERLVLRNFKVWDLSTCSMPLQAALGKDPVVS  417 

AtTPL_At1g15750    420 VNRVIWSPDGSLFGVAYSRHIVQLYSYHGGEDMRQHLEIDAHVGGVNDISFSTPNKQLCV  479 
                       VNRVIWSPDGSLFGVAYSRHIVQ+YSYHGG+D+RQHLEIDAHVGGVND++FS PNKQLCV      
HbTPL_7591         418 VNRVIWSPDGSLFGVAYSRHIVQIYSYHGGDDVRQHLEIDAHVGGVNDLAFSNPNKQLCV  477 

AtTPL_At1g15750    480 ITCGDDKTIKVWDAATGVKRHTFEGHEAPVYSVCPHYKENIQFIFSTALDGKIKAWLYDN  539 
                       ITCGDDKTIKVWDAATG K++TFEGHEAPVYSVCPHYKENIQFIFSTALDGKIKAWLYDN      
HbTPL_7591         478 ITCGDDKTIKVWDAATGTKQYTFEGHEAPVYSVCPHYKENIQFIFSTALDGKIKAWLYDN  537 

AtTPL_At1g15750    540 MGSRVDYDAPGRWCTTMAYSADGTRLFSCGTSKDGESFIVEWNESEGAVKRTYQGFHKRS  599 
                       +GSRVDY+APGRWCTTMAYSADGTRLFSCGTSK+GES IVEWNESEG VKR+Y GF KRS      
HbTPL_7591         538 LGSRVDYEAPGRWCTTMAYSADGTRLFSCGTSKEGESHIVEWNESEGNVKRSYLGFRKRS  597 

AtTPL_At1g15750    600 LGVVQFDTTKNRYLAAGDDFSIKFWDMDAVQLLTAIDGDGGLQASPRIRFNKEGSLLAVS  659 
                       LGVVQFDTTKNR+LAAGDDFSIKFWDMD VQLLT+ID DGGL ASPRIRFNK+G+LLAVS      
HbTPL_7591         598 LGVVQFDTTKNRFLAAGDDFSIKFWDMDNVQLLTSIDADGGLPASPRIRFNKDGTLLAVS  657 

AtTPL_At1g15750    660 GNENVIKIMANSDGLRLLHTFENISSESSKPA----------INSIAAAAAAAATSAGHA  709 
                        NEN IKI+ANSDGLRLL +FEN+S ++S+ +          I++ AAAAAAAATSAG A      
HbTPL_7591         658 ANENGIKILANSDGLRLLRSFENLSYDASRASETVTKPIISPISAAAAAAAAAATSAGLA  717 

AtTPL_At1g15750    710 DRSANVVSIQGMNGDSRNMVDVKPVITEESNDKSKIWKLTEVSEPSQCRSLRLPENLRVA  769 
                       DR+A++V+I GMNGD+RNM DVKP I EESNDKSKIWKLTE++EPSQCRSLRLPENLRV       
HbTPL_7591         718 DRNASMVTIPGMNGDARNMGDVKPRINEESNDKSKIWKLTEINEPSQCRSLRLPENLRVN  777 

AtTPL_At1g15750    770 KISRLIFTNSGNAILALASNAIHLLWKWQRNERNATGKATASLPPQQWQPASGILMTNDV  829 
                       KISRLI+TNSGNAILALASNAIHLLWKWQR++RN+TGKATA++ PQ WQP+SGILMTND+      
HbTPL_7591         778 KISRLIYTNSGNAILALASNAIHLLWKWQRSDRNSTGKATANVSPQLWQPSSGILMTNDI  837 

AtTPL_At1g15750    830 AETNPEEAVPCFALSKNDSYVMSASGGKISLFNMMTFKTMATFMPPPPAATFLAFHPQDN  889 
                        +TNPEEAVPCFALSKNDSYVMSASGGKISLFNMMTFKTM TFMPPPPAATFLAFHPQDN      
HbTPL_7591         838 TDTNPEEAVPCFALSKNDSYVMSASGGKISLFNMMTFKTMTTFMPPPPAATFLAFHPQDN  897 

AtTPL_At1g15750    890 NIIAIGMDDSTIQIYNVRVDEVKSKLKGHSKRITGLAFSNVLNVLVSSGADAQLCVWNTD  949 
                       NIIAIGMDDSTIQIYNVRVDEVKSKLKGHSKRITGLAFS+VLNVLVSSGADAQLCVWN+D      
HbTPL_7591         898 NIIAIGMDDSTIQIYNVRVDEVKSKLKGHSKRITGLAFSHVLNVLVSSGADAQLCVWNSD  957 

AtTPL_At1g15750    950 GWEKQRSKVLPLPQGRPNSAPSDTRVQFHQDQAHFLVVHETQLAIYETTKLE--CMKQWA 1007 
                       GWEKQ+++ L +P GR  +  SDTRVQFHQDQ  FLVVHETQLAIYE TKLE  C KQW       
HbTPL_7591         958 GWEKQKTRFLQVPPGRTTTGQSDTRVQFHQDQIQFLVVHETQLAIYEATKLECPCPKQWV 1017 

AtTPL_At1g15750   1008 VRESLAPITHATFSCDSQLVYASFMDATVCVFSSANLRLRCRVNPSAYLPASLSNSNVHP 1067 
                         ES API+HATFSCDSQLVYASF+DATV VFS+ NLRLRCR+NPS+YLPA++S+SNVHP      
HbTPL_7591        1018 TPESSAPISHATFSCDSQLVYASFLDATVRVFSAQNLRLRCRINPSSYLPANVSSSNVHP 1077 

AtTPL_At1g15750   1068 LVIAAHPQEPNMFAVGLSDGGVHIFEPLESEGKWGVAPPAENGSASGAPTAPSVGASASD 1127 
                       LVIAAHPQE N FA+GLSDGGVH+FEPLESEGKWGV PPAENGSAS A  AP VG S SD      
HbTPL_7591        1078 LVIAAHPQESNQFALGLSDGGVHVFEPLESEGKWGVPPPAENGSASSA--APPVGPSGSD 1135 

AtTPL_At1g15750   1128 QPQR- 1131 
                       Q QR       
HbTPL_7591        1136 QAQR* 1140
