# Supplementary material for: Transcriptional and post-transcriptional regulation of the jasmonate signalling pathway in response to abiotic and harvesting stress in Hevea brasiliensis
Source: BMC Plant Biol. 2014 Dec 2;14:341. doi: 10.1186/s12870-014-0341-0 (PMC4274682; doi:10.1186/s12870-014-0341-0)
Supplement: Additional file 11: — Amino acids sequence alignment of HbMED25 with AtMED25. [file 12870_2014_341_MOESM11_ESM.docx]

>Protein alignment 174 Alignment of 2 sequences: AtMED25_At1g25540, HbMED25_167
87

Identities = 434/855 (50%), Positives = 516/855 (60%), Gaps = 173/855 (20%)

AtMED25_At1g25540     1 MSSEVKQLIVVAEGTAALGPYWQTIVSDYLEKIIRSFCGSELNGERNPVSTVELSLVIFN  60 
                                                                                         
HbMED25_16787           ------------------------------------------------------------     

AtMED25_At1g25540    61 SHGSYCACLVQRSGWTRDVDIFLHWLSSIQFGGGGFNEVATAEGLAEALMMFSPPSGQAQ 120 
                                              L W  S                               Q     
HbMED25_16787         1 ---------------------MLQWKPS-------------------------------Q   8 

AtMED25_At1g25540   121 PSNDLKRHCILITASNPHILPTPVYRPRLQNVERNENGDAQAESRLSDAETVASYFAKCS 180 
                         + D +RHC+LI ASNP+ LPTPVYRP++QN+E++EN D Q ESRLSD+ETVA  F +CS     
HbMED25_16787         9 QNIDGQRHCVLIAASNPYPLPTPVYRPQIQNLEQSENIDQQTESRLSDSETVAKSFPQCS  68 

AtMED25_At1g25540   181 VSLSVVCPKQLPTIRALYNAGKPNQQSADLSIDTAKNTFYLVLISENFVEACAALSHSA- 239 
                        VSLSV+CPKQL  IRA+YNAGK N ++AD  +D  KN  +LVLISENF+EA A+LS         
HbMED25_16787        69 VSLSVICPKQLSKIRAIYNAGKRNIRAADPIVDNVKNPHFLVLISENFMEARASLSRPGV 128 

AtMED25_At1g25540   240 TNLPQTQSPVKVDRATVAPSIPVTGQPPAPVSSANGPIQNRQPVSVGPVPTATVKVEPST 299 
                         NLP  QSPVKVD A+V  +    G  P  + S NG I NR P+SV  VPTATVKVEP+T     
HbMED25_16787       129 ANLPSNQSPVKVDVASVTSA---AGPAPPSIPSVNGSILNRPPISVANVPTATVKVEPTT 185 

AtMED25_At1g25540   300 VTSMAPVPSFPHIPAVARPATQAIPSIQTSSASPVSQDMVSNAENAPDIKPVVVSGMTPP 359 
                        VTS+AP P+F HIP+ ARP +QA+PS+QTSS    +Q+M+++ EN PD+KP  VSGM        
HbMED25_16787       186 VTSIAPGPAFSHIPS-ARPTSQAVPSLQTSSPPTSTQEMIASGENVPDLKP-SVSGMPQS 243 

AtMED25_At1g25540   360 LRTGPPGGANVNLLNNLSQVRQVMSSAALAGAAS----SVGQSAVAMHMSNMISTGMATS 415 
                        +R  PPG ANV++LNNLSQ RQVM+SAAL G  +    S+ Q+ VAMHMSNMIS+GMA+S     
HbMED25_16787       244 VRPVPPGAANVSILNNLSQARQVMNSAALTGGTTIGLQSMNQTPVAMHMSNMISSGMASS 303 

AtMED25_At1g25540   416 LPPSQTVFSTGQQGITSMAGSGALMGSAQTGQSPGPNNAFSPQTTSNVASNLGVSQPM-- 473 
                        +PP+Q VFS+GQ GI+S+ GS    G+ Q   + G  +  S  +  +  SNLG+SQPM       
HbMED25_16787       304 VPPAQNVFSSGQPGISSITGS----GTTQIASNSGLGSFTSATSNISGNSNLGISQPMGN 359 

AtMED25_At1g25540   474 -----------QGMNQGSHSGA-MMQGGISMNQNMMSGLGQGNVSSGTGGMMPTPGVGQQ 521 
                                    GM+QG+ SGA M+Q GISMNQNMMSGLG   VSSG+  M+PTPG+ QQ     
HbMED25_16787       360 LQGAVSIGPSVPGMSQGNLSGAQMVQSGISMNQNMMSGLGPSGVSSGSSTMIPTPGMPQQ 419 

AtMED25_At1g25540   522 AQSGIQQLGGSNSSAPNMQLSQPSSGAMQTSQSKYVKVWEGNLSGQRQGQPVLITRLEGY 581 
                        AQSG+Q LG +N+SA NM L Q ++ A+Q++QSKYVKVWEGNLSGQRQGQPV ITRLEGY     
HbMED25_16787       420 AQSGMQTLGVNNNSAANMPLPQQTTSALQSAQSKYVKVWEGNLSGQRQGQPVFITRLEGY 479 

AtMED25_At1g25540   582 RSASASDSLAANWPPTMQIVRLISQDHMNNKQYVGKADFLVFRAMSQHGFLGQLQDKKLC 641 
                        RSA A + LA+NWPPTMQIVRLISQDHMNNKQYVGKADFLVFRAM+QHGFLGQLQ+KKLC     
HbMED25_16787       480 RSAVAPEDLASNWPPTMQIVRLISQDHMNNKQYVGKADFLVFRAMNQHGFLGQLQEKKLC 539 

AtMED25_At1g25540   642 AVIQLPSQTLLLSVSDKACRLIGMLFPGDMVVFKPQIPNQQQQQQQQLHQQQQQQQQIQQ 701 
                        AVIQLPSQTLLLSVSDKA RLIGMLFPGDMVVFKPQI +QQQQ QQQ HQ        Q      
HbMED25_16787       540 AVIQLPSQTLLLSVSDKAFRLIGMLFPGDMVVFKPQISSQQQQMQQQHHQ--------QM 591 

AtMED25_At1g25540   702 QQQQQQHLQQQQMPQLQQQQQQHQQQQQQQHQLSQLQHHQQQQQQQQQQQQQHQLTQLQH 761 
                          QQ   LQQQQ+PQLQQQQQ  Q QQQQ  QL QLQH    QQQ  Q QQQ QL+QLQ      
HbMED25_16787       592 PTQQHPQLQQQQLPQLQQQQQVPQLQQQQ--QLPQLQH----QQQHPQLQQQQQLSQLQ- 644 

AtMED25_At1g25540   762 HHQQQQQASPLNQMQQQTSPLNQMQQQTSPLNQMQQQQQPQQMVMGGQAFAQAPGRSQQG 821 
                          QQQQ+ +  N                S  +          +     A A A   S        
HbMED25_16787       645 --QQQQRTTDFN----------------SSSSSFHSCSSSSAVAAAAAASAVAAAASAVA 686 

AtMED25_At1g25540   822 GGGGQPNMPGAGFMG 836 
                               +  A         
HbMED25_16787       687 AAAAASAVAAATSTA 701
