# Supplementary material for: Transcriptional and post-transcriptional regulation of the jasmonate signalling pathway in response to abiotic and harvesting stress in Hevea brasiliensis
Source: BMC Plant Biol. 2014 Dec 2;14:341. doi: 10.1186/s12870-014-0341-0 (PMC4274682; doi:10.1186/s12870-014-0341-0)
Supplement: Additional file 12: — Analysis of variance of gene expression according the tested tissues. [file 12870_2014_341_MOESM12_ESM.docx]

Variable: XCOI_3058

Df Sum Sq Mean Sq F value Pr(>F)

Tissu 10 4.781 0.4781 11.39 1.43e-06 ***

Residuals 22 0.923 0.0420

---

Signif. codes: 0 ‘***’ 0.001 ‘**’ 0.01 ‘*’ 0.05 ‘.’ 0.1 ‘ ’ 1

Groups, Treatments and means

a Latex 0.7622

a Roots 3 0.7115

ab Fem imm 0.6217

abc male imm 0.5661

abcd male mat 0.4602

abcd Roots 1 0.4205

abcd Leaf 0.1734

bcd Fem mat 0.08479

cde MF cotyl 0.002926

de Bark -0.04571

e MFE body -0.5525

<<<<<<<<<<<<<<<<<<<<<<<<<<<<<<<<<<<<<<<<<<<

Variable: XCOI_2304

Df Sum Sq Mean Sq F value Pr(>F)

Tissu 10 2.898 0.28980 5.969 0.000238 ***

Residuals 22 1.068 0.04855

---

Signif. codes: 0 ‘***’ 0.001 ‘**’ 0.01 ‘*’ 0.05 ‘.’ 0.1 ‘ ’ 1

Groups, Treatments and means

a Latex 0.5556

ab Roots 3 0.2644

abc Roots 1 0.2234

abc Leaf -0.04617

bc Fem imm -0.1428

bc male imm -0.2435

bc MF cotyl -0.2906

bc Bark -0.3044

bc Fem mat -0.3056

bc MFE body -0.3482

c male mat -0.403

<<<<<<<<<<<<<<<<<<<<<<<<<<<<<<<<<<<<<<<<<<

Variable: XJAZ_2001

Df Sum Sq Mean Sq F value Pr(>F)

Tissu 10 2.0849 0.20849 8.166 2.27e-05 ***

Residuals 22 0.5617 0.02553

---

Signif. codes: 0 ‘***’ 0.001 ‘**’ 0.01 ‘*’ 0.05 ‘.’ 0.1 ‘ ’ 1

Groups, Treatments and means

a male imm 0.8355

ab Fem imm 0.5172

ab MF cotyl 0.4382

abc male mat 0.4261

abcd Roots 3 0.3777

abcd Fem mat 0.3765

bcd Bark 0.2249

bcd Roots 1 0.131

bcd Latex 0.1208

cd Leaf -0.02944

d MFE body -0.07355

<<<<<<<<<<<<<<<<<<<<<<<<<<<<<<<<<<<<<

Variable: XJAZ_1660

Df Sum Sq Mean Sq F value Pr(>F)

Tissu 10 11.887 1.1887 21.36 4.48e-09 ***

Residuals 22 1.224 0.0556

---

Signif. codes: 0 ‘***’ 0.001 ‘**’ 0.01 ‘*’ 0.05 ‘.’ 0.1 ‘ ’ 1

Groups, Treatments and means

a Latex 0.6143

ab Fem imm 0.4855

abc male imm 0.3349

bcd Fem mat -0.1131

bcd Bark -0.1648

cd Roots 3 -0.2406

d Roots 1 -0.3654

de Leaf -0.6336

de male mat -0.7006

ef MF cotyl -1.064

f MFE body -1.391

<<<<<<<<<<<<<<<<<<<<<<<<<<<<<<<<<<<<<<<<<<<<<<<<<<<<

Variable: XJAZ_1405

Df Sum Sq Mean Sq F value Pr(>F)

Tissu 10 33.28 3.328 32.18 7.9e-11 ***

Residuals 22 2.28 0.103

---

Signif. codes: 0 ‘***’ 0.001 ‘**’ 0.01 ‘*’ 0.05 ‘.’ 0.1 ‘ ’ 1

Groups, Treatments and means

a male mat 0.926

ab Latex 0.4856

abc Fem mat 0.1475

bcd Bark -0.07704

cde Fem imm -0.5407

cde male imm -0.6062

de MF cotyl -0.8177

ef MFE body -1.017

ef Roots 3 -1.467

fg Roots 1 -1.899

g Leaf -2.64

<<<<<<<<<<<<<<<<<<<<<<<<<<<<<<<<<<<<<<<<<<<<

Variable: XJAZ_1229

Df Sum Sq Mean Sq F value Pr(>F)

Tissu 10 10.55 1.0553 20.01 8.39e-09 ***

Residuals 22 1.16 0.0527

---

Signif. codes: 0 ‘***’ 0.001 ‘**’ 0.01 ‘*’ 0.05 ‘.’ 0.1 ‘ ’ 1

Groups, Treatments and means

a Fem imm 0.8993

ab male imm 0.4832

ab Bark 0.3758

abc Fem mat 0.2432

bc Latex -0.08017

bcd male mat -0.1091

bcd Leaf -0.1519

cde Roots 3 -0.3633

de Roots 1 -0.7598

e MFE body -0.8786

e MF cotyl -0.951

<<<<<<<<<<<<<<<<<<<<<<<<<<<<<<<<<<<<<<<<<<<

Variable: XJAZ_863

Df Sum Sq Mean Sq F value Pr(>F)

Tissu 10 9.982 0.9982 72.75 1.71e-14 ***

Residuals 22 0.302 0.0137

---

Signif. codes: 0 ‘***’ 0.001 ‘**’ 0.01 ‘*’ 0.05 ‘.’ 0.1 ‘ ’ 1

Groups, Treatments and means

a Fem imm 1.397

b Bark 1.025

b male imm 1.01

b Fem mat 0.9607

bc Roots 3 0.8641

bc Latex 0.7869

bc Leaf 0.7118

c male mat 0.5763

d Roots 1 -0.08387

d MFE body -0.2089

d MF cotyl -0.3871

<<<<<<<<<<<<<<<<<<<<<<<<<<<<<<<<<<<<<<<<<<<<

Variable: XMYC_771

Df Sum Sq Mean Sq F value Pr(>F)

Tissu 10 17.062 1.7062 40.89 6.92e-12 ***

Residuals 22 0.918 0.0417

---

Signif. codes: 0 ‘***’ 0.001 ‘**’ 0.01 ‘*’ 0.05 ‘.’ 0.1 ‘ ’ 1

Groups, Treatments and means

a Latex 0.8946

b MF cotyl -0.111

bc MFE body -0.4919

bcd male mat -0.6116

bcd Bark -0.6183

bcd Roots 1 -0.6577

cde Fem mat -0.9389

de Fem imm -1.173

e male imm -1.303

e Roots 3 -1.361

f Leaf -2.003

<<<<<<<<<<<<<<<<<<<<<<<<<<<<<<<<<<<<<<<<

Variable: XNINJA_6328

Df Sum Sq Mean Sq F value Pr(>F)

Tissu 10 2.143 0.21430 2.381 0.0431 *

Residuals 22 1.980 0.08998

---

Signif. codes: 0 ‘***’ 0.001 ‘**’ 0.01 ‘*’ 0.05 ‘.’ 0.1 ‘ ’ 1

Groups, Treatments and means

a Roots 1 -2.011

a MFE body -2.044

a MF cotyl -2.055

a Roots 3 -2.137

a male imm -2.178

a male mat -2.191

a Fem imm -2.256

a Latex -2.339

a Fem mat -2.446

a Bark -2.758

a Leaf -2.768

<<<<<<<<<<<<<<<<<<<<<<<<<<<<<<<<<<<<<<<<

Variable: XJAR_5108

Df Sum Sq Mean Sq F value Pr(>F)

Tissu 10 29.547 2.9547 38.52 1.28e-11 ***

Residuals 22 1.688 0.0767

---

Signif. codes: 0 ‘***’ 0.001 ‘**’ 0.01 ‘*’ 0.05 ‘.’ 0.1 ‘ ’ 1

Groups, Treatments and means

a male imm 1.617

ab male mat 1.139

ab Fem imm 1.097

b Roots 3 0.5702

bc Fem mat 0.4235

bc Bark 0.4003

cd Roots 1 -0.2914

cd Leaf -0.3232

de MF cotyl -0.5638

ef MFE body -1.291

f Latex -1.435

<<<<<<<<<<<<<<<<<<<<<<<<<<<<<<<<<<<<

Variable: XMYC_424

Df Sum Sq Mean Sq F value Pr(>F)

Tissu 10 23.785 2.3785 79.59 6.59e-15 ***

Residuals 22 0.657 0.0299

---

Signif. codes: 0 ‘***’ 0.001 ‘**’ 0.01 ‘*’ 0.05 ‘.’ 0.1 ‘ ’ 1

Groups, Treatments and means

a Latex 1.039

b Bark -0.2596

bc MF cotyl -0.448

cd MFE body -0.7819

de Roots 1 -1.083

def male mat -1.206

def Fem imm -1.275

ef Fem mat -1.305

ef Roots 3 -1.365

f male imm -1.669

g Leaf -2.44

<<<<<<<<<<<<<<<<<<<<<<<<<<<<<<<<<<<<<

Variable: XTPL_7591

Df Sum Sq Mean Sq F value Pr(>F)

Tissu 10 7.899 0.7899 9.367 7.49e-06 ***

Residuals 22 1.855 0.0843

---

Signif. codes: 0 ‘***’ 0.001 ‘**’ 0.01 ‘*’ 0.05 ‘.’ 0.1 ‘ ’ 1

Groups, Treatments and means

a MFE body -1.601

ab MF cotyl -1.931

ab Fem mat -2.153

ab Leaf -2.237

ab male imm -2.246

abc Roots 1 -2.321

abc Fem imm -2.321

bcd Latex -2.549

bcd Bark -2.71

cd male mat -3.161

d Roots 3 -3.378

<<<<<<<<<<<<<<<<<<<<<<<<<<<<<<<<<<<<

Variable: XJAZ_14313

Df Sum Sq Mean Sq F value Pr(>F)

Tissu 10 0.9320 0.09320 7.076 6.86e-05 ***

Residuals 22 0.2898 0.01317

---

Signif. codes: 0 ‘***’ 0.001 ‘**’ 0.01 ‘*’ 0.05 ‘.’ 0.1 ‘ ’ 1

Groups, Treatments and means

a Latex 1.953

a Bark 1.95

ab male imm 1.817

ab Roots 3 1.803

ab Fem mat 1.782

ab male mat 1.756

ab Fem imm 1.731

abc MFE body 1.688

bc Leaf 1.576

bc MF cotyl 1.552

c Roots 1 1.357

<<<<<<<<<<<<<<<<<<<<<<<<<<<<<<<<<<<<<<<<<

Variable: XJAR_14894

Df Sum Sq Mean Sq F value Pr(>F)

Tissu 10 6.812 0.6812 12.35 7.11e-07 ***

Residuals 22 1.213 0.0552

---

Signif. codes: 0 ‘***’ 0.001 ‘**’ 0.01 ‘*’ 0.05 ‘.’ 0.1 ‘ ’ 1

Groups, Treatments and means

a male mat 0.8381

a male imm 0.5702

ab Fem imm 0.3866

ab Bark 0.3536

abc Roots 3 0.2806

abcd Fem mat 0.1727

bcde Leaf -0.2034

bcde Latex -0.2144

cde MF cotyl -0.3406

de MFE body -0.4321

e Roots 1 -0.7168

<<<<<<<<<<<<<<<<<<<<<<<<<<<<<<<<<<<<<<<<<

Variable: XMED25_16787

Df Sum Sq Mean Sq F value Pr(>F)

Tissu 10 1.0149 0.10149 5.102 7e-04 ***

Residuals 22 0.4376 0.01989

---

Signif. codes: 0 ‘***’ 0.001 ‘**’ 0.01 ‘*’ 0.05 ‘.’ 0.1 ‘ ’ 1

Groups, Treatments and means

a MF cotyl 1.419

ab male imm 1.265

abc MFE body 1.198

abc Fem mat 1.161

abc Fem imm 1.109

abc Roots 3 1.03

bc male mat 0.9883

bc Leaf 0.9567

bc Roots 1 0.9148

c Latex 0.8505

c Bark 0.8266

<<<<<<<<<<<<<<<<<<<<<<<<<<<<<<<<<<<<<

Variable: XJAZ_17062

Df Sum Sq Mean Sq F value Pr(>F)

Tissu 10 5.130 0.5130 8.644 1.44e-05 ***

Residuals 22 1.306 0.0593

---

Signif. codes: 0 ‘***’ 0.001 ‘**’ 0.01 ‘*’ 0.05 ‘.’ 0.1 ‘ ’ 1

Groups, Treatments and means

a MFE body -0.2676

a Roots 3 -0.2991

a MF cotyl -0.3531

a Latex -0.3548

ab Roots 1 -0.5364

ab Bark -0.6445

ab Leaf -0.7355

ab male mat -0.7696

bc Fem mat -1.18

bc Fem imm -1.183

c male imm -1.49

<<<<<<<<<<<<<<<<<<<<<<<<<<<<<<<<<<

Variable: XJAZ_19967

Df Sum Sq Mean Sq F value Pr(>F)

Tissu 10 21.138 2.1138 116.1 <2e-16 ***

Residuals 22 0.401 0.0182

---

Signif. codes: 0 ‘***’ 0.001 ‘**’ 0.01 ‘*’ 0.05 ‘.’ 0.1 ‘ ’ 1

Groups, Treatments and means

a Fem imm 2.196

ab male mat 1.844

ab male imm 1.819

b Fem mat 1.753

c Bark 1.358

d MFE body 0.7364

de Latex 0.6075

de Leaf 0.6

ef Roots 3 0.294

fg MF cotyl -0.01925

g Roots 1 -0.2718

<<<<<<<<<<<<<<<<<<<<<<<<<<<<<<<<<<<<<<<<<<

Variable: XJAR_20244

Df Sum Sq Mean Sq F value Pr(>F)

Tissu 10 14.603 1.4603 9.792 5.19e-06 ***

Residuals 22 3.281 0.1491

---

Signif. codes: 0 ‘***’ 0.001 ‘**’ 0.01 ‘*’ 0.05 ‘.’ 0.1 ‘ ’ 1

Groups, Treatments and means

a male imm -0.4616

ab Fem imm -0.6978

abc Leaf -1.249

abcd Fem mat -1.39

abcd male mat -1.402

abcd MF cotyl -1.431

abcd MFE body -1.445

bcd Roots 3 -1.754

cde Bark -1.912

de Latex -2.403

e Roots 1 -2.926

<<<<<<<<<<<<<<<<<<<<<<<<<<<<<<<<<<<<<<

Variable: XJAR_20347

Df Sum Sq Mean Sq F value Pr(>F)

Tissu 10 1.570 0.15699 2.871 0.0188 *

Residuals 22 1.203 0.05468

---

Signif. codes: 0 ‘***’ 0.001 ‘**’ 0.01 ‘*’ 0.05 ‘.’ 0.1 ‘ ’ 1

Groups, Treatments and means

a male mat -3.759

ab Bark -3.826

ab Roots 1 -3.828

ab male imm -3.87

ab Latex -3.876

ab Fem imm -3.921

ab Roots 3 -3.96

ab Fem mat -4.046

ab MFE body -4.089

ab MF cotyl -4.354

b Leaf -4.48

<<<<<<<<<<<<<<<<<<<<<<<<<<<<<<<<<<<<<<<

Variable: XJAR_21367

Df Sum Sq Mean Sq F value Pr(>F)

Tissu 10 40.53 4.053 144.4 <2e-16 ***

Residuals 22 0.62 0.028

---

Signif. codes: 0 ‘***’ 0.001 ‘**’ 0.01 ‘*’ 0.05 ‘.’ 0.1 ‘ ’ 1

Groups, Treatments and means

a male mat -0.4494

ab male imm -0.7571

bc Fem imm -1.112

c Fem mat -1.379

c Roots 3 -1.492

d MFE body -2.184

e MF cotyl -2.872

e Roots 1 -2.935

e Bark -3.014

e Leaf -3.102

f Latex -4.096

<<<<<<<<<<<<<<<<<<<<<<<<<<<<<<<<<<<<<<<<

Variable: XJAZ_26925

Df Sum Sq Mean Sq F value Pr(>F)

Tissu 10 1.695 0.16946 10.19 3.73e-06 ***

Residuals 22 0.366 0.01664

---

Signif. codes: 0 ‘***’ 0.001 ‘**’ 0.01 ‘*’ 0.05 ‘.’ 0.1 ‘ ’ 1

Groups, Treatments and means

a male imm 3.145

ab Fem imm 3.056

ab Fem mat 3.045

abc male mat 3.023

abc Roots 3 2.973

abc Bark 2.902

abcd Leaf 2.773

bcd MFE body 2.73

cd MF cotyl 2.665

d Roots 1 2.51

d Latex 2.425

<<<<<<<<<<<<<<<<<<<<<<<<<<<<<<<<<<<<<<<<

Variable: XJAZ_29511

Df Sum Sq Mean Sq F value Pr(>F)

Tissu 10 15.522 1.5522 44.86 2.67e-12 ***

Residuals 22 0.761 0.0346

---

Signif. codes: 0 ‘***’ 0.001 ‘**’ 0.01 ‘*’ 0.05 ‘.’ 0.1 ‘ ’ 1

Groups, Treatments and means

a Fem mat -0.4669

a Fem imm -0.5937

ab male imm -0.6748

abc Latex -0.9948

bc male mat -1.151

cd Roots 3 -1.395

de Bark -1.841

de MFE body -1.875

ef Roots 1 -2.174

ef Leaf -2.215

f MF cotyl -2.514

<<<<<<<<<<<<<<<<<<<<<<<<<<<<<<<<<<<<<<

Variable: XJAR_59958

Df Sum Sq Mean Sq F value Pr(>F)

Tissu 10 72.57 7.257 8.824 1.22e-05 ***

Residuals 22 18.09 0.822

---

Signif. codes: 0 ‘***’ 0.001 ‘**’ 0.01 ‘*’ 0.05 ‘.’ 0.1 ‘ ’ 1

Groups, Treatments and means

a Roots 1 -1.424

a Roots 3 -1.446

a Latex -1.511

ab MF cotyl -1.997

ab MFE body -2.1

ab Bark -2.106

abc Leaf -3.507

abc Fem imm -3.646

bc male imm -4.381

c Fem mat -4.768

c male mat -5.959

<<<<<<<<<<<<<<<<<<<<<<<

Variable: XMYC_94937

Df Sum Sq Mean Sq F value Pr(>F)

Tissu 10 12.092 1.2092 9.55 6.38e-06 ***

Residuals 22 2.786 0.1266

---

Signif. codes: 0 ‘***’ 0.001 ‘**’ 0.01 ‘*’ 0.05 ‘.’ 0.1 ‘ ’ 1

Groups, Treatments and means

a Latex -2.271

ab MF cotyl -2.43

ab MFE body -2.689

abc Roots 3 -3.105

abcd Fem imm -3.284

abcd Fem mat -3.3

bcd Roots 1 -3.45

cd Bark -3.736

cd male mat -3.759

cd Leaf -3.999

d male imm -4.244
