# Supplementary material for: Transcriptional and post-transcriptional regulation of the jasmonate signalling pathway in response to abiotic and harvesting stress in Hevea brasiliensis
Source: BMC Plant Biol. 2014 Dec 2;14:341. doi: 10.1186/s12870-014-0341-0 (PMC4274682; doi:10.1186/s12870-014-0341-0)
Supplement: Additional file 13: — Analysis of variance (ANOVA) to test effect of tapping,ethephon treatment, tissue and cross effects on the expression of the different gene of the JA signaling pathway. [file 12870_2014_341_MOESM13_ESM.docx]

**Additional file 11 :** Analysis of variance (ANOVA) to test effect of tapping, ethephon treatment, tissue and cross effects on the expression of the different gene of the JA signaling pathway

| **Gene** | **Variable** | **df** | **Sum Sq** | **Mean Sq** | **F value** | **P (>F)** |
| --- | --- | --- | --- | --- | --- | --- |
| **COI_2304** | Tissue  Tapping  Ethephon  Tissue x Tapping  Tissue x Ethephon  Tapping x Ethephon  Tissue x Tapping x Ethephon  Residuals | 1  1  1  1  1  1  1  16 | 1.1688  0.0353  0.0007  0.5556  0.0003  0.0007  0.0058  1.0042 | 1.1688  0.0353  0.0007  0.5556  0.0003  0.0007  0.0058  0.0628 | 18.624  0.562  0.011  8.853  0.005  0.011  0.092 | 0.000533  0.464185  0.918063  0.008926  0.945582  0.915938  0.765533 |
| **COI_3058** | Tissue  Tapping  Ethephon  Tissue x Tapping  Tissue x Ethephon  Tapping x Ethephon  Tissue x Tapping x Ethephon  Residuals | 1  1  1  1  1  1  1  16 | 0.1427  0.0124  0.9666  0.0008  0.0070  0.5033  0.0054  0.7098 | 0.1427  0.0124  0.9666  0.0008  0.0070  0.5033  0.0054  0.0444 | 3.271  0.279  21.788  0.017  0.158  11.346  0.122 | 0.091819  0.604633  0.000257  0.897729  0.696052  0.003912  0.731287 |
| **JAZ_863** | Tissue  Tapping  Ethephon  Tissue x Tapping  Tissue x Ethephon  Tapping x Ethephon  Tissue x Tapping x Ethephon  Residuals | 1  1  1  1  1  1  1  16 | 0.0243  0.7654  0.5835  0.3582  2.0388  0.1959  0.4369  0.4212 | 0.0243  0.7654  0.5835  0.3582  2.0388  0.1959  0.4369  0.0263 | 0.922  29.074  22.162  13.604  77.441  7.442  16.596 | 0.351202  5.99^e^-05  0.000237  0.001991  1.58^e^-07  0.014893  0.000884 |
| **JAZ_14313** | Tissue  Tapping  Ethephon  Tissue x Tapping  Tissue x Ethephon  Tapping x Ethephon  Tissue x Tapping x Ethephon  Residuals | 1  1  1  1  1  1  1  16 | 0.0124  0.2490  1.6062  0.0032  0.5623  0.0105  0.1022  1.0966 | 0.0124  0.2490  1.6062  0.0032  0.5623  0.0105  0.1022  0.0685 | 0.181  3.632  23.434  0.047  8.204  0.154  1.492 | 0.676359  0.074792  0.000181  0.830778  0.011246  0.700148  0.239657 |
| **JAZ_1229** | Tissue  Tapping  Ethephon  Tissue x Tapping  Tissue x Ethephon  Tapping x Ethephon  Tissue x Tapping x Ethephon  Residuals | 1  1  1  1  1  1  1  16 | 0.3683  0.0060  0.4524  2.5951  1.2118  0.1106  1.1650  0.5345 | 0.3683  0.0060  0.4524  2.5951  1.2118  0.1106  1.1650  0.0334 | 11.024  0.179  13.542  77.682  36.273  3.309  34.873 | 0.00433  0.67775  0.00203  1.54^e^-07  1.77^e^-05  0.08765  2.22^e^-05 |
| **JAZ_2001** | Tissue  Tapping  Ethephon  Tissue x Tapping  Tissue x Ethephon  Tapping x Ethephon  Tissue x Tapping x Ethephon  Residuals | 1  1  1  1  1  1  1  16 | 5.369  0.313  0.681  0.487  0.005  0.204  0  0.499 | 5.369  0.313  0.681  0.487  0.005  0.204  0  0.031 | 172.298  10.05  21.864  15.634  0.164  6.533  0.010 | 5.55^e^-10  0.005939  0.000253  0.001137  0.691115  0.021148  0.921409 |
| **JAZ_19967** | Tissue  Tapping  Ethephon  Tissue x Tapping  Tissue x Ethephon  Tapping x Ethephon  Tissue x Tapping x Ethephon  Residuals | 1  1  1  1  1  1  1  16 | 23.43  64.05  29.92  17.91  37.16  42.70  21.01  0.86 | 23.43  64.05  29.92  17.91  37.16  42.70  21.01  0.05 | 436.0  1192.1  556.9  333.3  691.5  794.7  391.0 | 4.92^e^-13  <2^e^-16  7.36^e^-14  3.88^e^-121.36^e^-14  4.56^e^-15  1.14^e^-12 |
| **JAZ_1660** | Tissue  Tapping  Ethephon  Tissue x Tapping  Tissue x Ethephon  Tapping x Ethephon  Tissue x Tapping x Ethephon  Residuals | 1  1  1  1  1  1  1  16 | 1.7259  0.3830  2.2254  0.0003  0.0005  0.6631  0.0873  0.4347 | 1.7259  0.3830  2.2254  0.0003  0.0005  0.6631  0.0873  0.0272 | 63.528  14.096  81.914  0.011  0.018  24.407  3.214 | 5.82^e^-07  0.001731  1.08^e^-07  0.919196  0.896364  0.000148  0.091945 |
| **JAZ_17062** | Tissue  Tapping  Ethephon  Tissue x Tapping  Tissue x Ethephon  Tapping x Ethephon  Tissue x Tapping x Ethephon  Residuals | 1  1  1  1  1  1  1  16 | 26.488  21.159  15.197  30.086  27.205  21.571  29.097  0.555 | 26.488  21.159  15.197  30.086  27.205  21.571  29.097  0.035 | 764.1  610.4  438.4  867.9  784.8  622.2  839.3 | 6.21^e^-15  3.60^e^-14  4.72^e^-13  2.28^e^-15  5.03^e^-15  3.10^e^-14  2.87^e^-15 |
| **JAZ_29511** | Tissue  Tapping  Ethephon  Tissue x Tapping  Tissue x Ethephon  Tapping x Ethephon  Tissue x Tapping x Ethephon  Residuals | 1  1  1  1  1  1  1  16 | 10.19  30.70  13.83  21.24  31.79  16.90  21.15  0.91 | 10.19  30.70  13.83  21.24  31.79  16.90  21.15  0.06 | 178.2  537.2  242.0  371.6  556.2  295.7  370.1 | 4.33^e^-10  9.75^e^-14  4.43^e^-11  1.69^e^-12  7.43^e^-14  9.68^e^-12  1.74^e^-12 |
| **JAZ_26925** | Tissue  Tapping  Ethephon  Tissue x Tapping  Tissue x Ethephon  Tapping x Ethephon  Tissue x Tapping x Ethephon  Residuals | 1  1  1  1  1  1  1  16 | 0  0.8  171.85  208.87  0.03  1.14  215.23  0.49 | 0  0.8  171.85  208.87  0.03  1.14  215.23 | 0.005  26.070  5619.467  6829.824  0.886  37.400  7037.776 | 0.946214  0.000106  <2^e^-16  <2^e^-16  0.36623  1.49^e^-05  <2^e^-16 |
| **JAZ_1405** | Tissue  Tapping  Ethephon  Tissue x Tapping  Tissue x Ethephon  Tapping x Ethephon  Tissue x Tapping x Ethephon  Residuals | 1  1  1  1  1  1  1  16 | 39.99  32.01  13.44  21.35  19.36  14.51  28.62  8.62 | 39.99  32.01  13.44  21.35  19.36  14.51  28.62  0.54 | 74.23  59.42  24.94  39.62  35.94  26.93  53.12 | 2.09^e^-07  8.95^e^-07  0.000133  1.07^e^-05  1.87^e^-05  8.95^e^-05  1.82^e^-06 |
| **MYC_771** | Tissue  Tapping  Ethephon  Tissue x Tapping  Tissue x Ethephon  Tapping x Ethephon  Tissue x Tapping x Ethephon  Residuals | 1  1  1  1  1  1  1  16 | 17.039  0.006  1.969  2.602  0.008  0.163  0.623  1.949 | 17.039  0.006  1.969  2.602  0.008  0.163  0.623  0.122 | 139.896  0.050  16.166  21.636  0.068  1.341  5.115 | 2.54^e^-09  0.825581  0.000988  0.000283  0.797182  0.263932  0.037995 |
| **MYC_94937** | Tissue  Tapping  Ethephon  Tissue x Tapping  Tissue x Ethephon  Tapping x Ethephon  Tissue x Tapping x Ethephon  Residuals | 1  1  1  1  1  1  1  16 | 32.07  11.46  4.5  5.18  0.00  3.53  1.17  0.74 | 32.07  11.46  4.5  5.18  0.00  3.53  1.17  0.05 | 691.39  247.09  97.06  111.78  0.09  76.21  25.17 | 1.36^e^-14  3.78^e^-11  3.38^e^-08  1.26^e^-08  0.768098  1.75^e^-07  0.000126 |
| **MYC_424** | Tissue  Tapping  Ethephon  Tissue x Tapping  Tissue x Ethephon  Tapping x Ethephon  Tissue x Tapping x Ethephon  Residuals | 1  1  1  1  1  1  1  16 | 20.175  0.137  2.252  3.359  0.034  0.068  0.422  1.804 | 20.175  0.137  2.252  3.359  0.034  0.068  0.422  0.113 | 178.893  1.218  19.971  29.784  0.303  0.600  3.740 | 4.21e-10  0.286126  0.000388  5.27e-05  0.589858  0.450012  0.071016 |
