# Supplementary material for: Transcriptional and post-transcriptional regulation of the jasmonate signalling pathway in response to abiotic and harvesting stress in Hevea brasiliensis
Source: BMC Plant Biol. 2014 Dec 2;14:341. doi: 10.1186/s12870-014-0341-0 (PMC4274682; doi:10.1186/s12870-014-0341-0)
Supplement: Additional file 14: — Analysis of variance of gene expression according the tested clone. [file 12870_2014_341_MOESM14_ESM.docx]

**Variable: MYC_424**

Df Sum Sq Mean Sq F value Pr(>F)

clone 2 0.0765 0.03826 1.268 0.31

Residuals 15 0.4527 0.03018

Groups, Treatments and means

a PB 217 0.4985

a RRIM 600 0.4125

a PB 260 0.3389

**Variable: MYC_771**

Df Sum Sq Mean Sq F value Pr(>F)

clone 2 1.6091 0.8045 14.75 0.000287 ***

Residuals 15 0.8179 0.0545

---

Signif. codes: 0 ‘***’ 0.001 ‘**’ 0.01 ‘*’ 0.05 ‘.’ 0.1 ‘ ’ 1

Study:

Groups, Treatments and means

a RRIM 600 -0.06876

a PB 217 -0.2119

b PB 260 -0.7624

**Variable: JAZ_863**

Df Sum Sq Mean Sq F value Pr(>F)

clone 2 0.2409 0.12046 3.191 0.07 .

Residuals 15 0.5663 0.03775

---

Signif. codes: 0 ‘***’ 0.001 ‘**’ 0.01 ‘*’ 0.05 ‘.’ 0.1 ‘ ’ 1

Groups, Treatments and means

a RRIM 600 -0.1542

a PB 260 -0.2558

a PB 217 -0.4341

**Variable: JAZ_1229**

Df Sum Sq Mean Sq F value Pr(>F)

clone 2 3.694 1.8468 29.36 6.52e-06 ***

Residuals 15 0.944 0.0629

---

Signif. codes: 0 ‘***’ 0.001 ‘**’ 0.01 ‘*’ 0.05 ‘.’ 0.1 ‘ ’ 1

Groups, Treatments and means

a PB 260 -0.2802

b RRIM 600 -0.9488

c PB 217 -1.381

**Variable: JAZ_1405**

Df Sum Sq Mean Sq F value Pr(>F)

clone 2 1.1476 0.5738 10.66 0.00132 **

Residuals 15 0.8076 0.0538

---

Signif. codes: 0 ‘***’ 0.001 ‘**’ 0.01 ‘*’ 0.05 ‘.’ 0.1 ‘ ’ 1

Groups, Treatments and means

a PB 260 -0.2475

b RRIM 600 -0.6694

b PB 217 -0.8501

**Variable: JAZ_1660**

Df Sum Sq Mean Sq F value Pr(>F)

clone 2 0.06564 0.03282 1.802 0.199

Residuals 15 0.27322 0.01821

Groups, Treatments and means

a RRIM 600 -0.3136

a PB 260 -0.4246

a PB 217 -0.4538

**Variable: JAZ_2001**

Df Sum Sq Mean Sq F value Pr(>F)

clone 2 0.3339 0.16695 1.779 0.203

Residuals 15 1.4078 0.09385

Groups, Treatments and means

a RRIM 600 -1.615

a PB 260 -1.72

a PB 217 -1.942

**Variable: COI_2304**

Df Sum Sq Mean Sq F value Pr(>F)

clone 2 0.1190 0.05949 1.336 0.292

Residuals 15 0.6678 0.04452

Groups, Treatments and means

a PB 260 -0.7072

a RRIM 600 -0.8107

a PB 217 -0.9063

**Variable: COI_3058**

Df Sum Sq Mean Sq F value Pr(>F)

clone 2 0.3944 0.19720 3.382 0.0613 .

Residuals 15 0.8747 0.05831

---

Signif. codes: 0 ‘***’ 0.001 ‘**’ 0.01 ‘*’ 0.05 ‘.’ 0.1 ‘ ’ 1

Groups, Treatments and means

a RRIM 600 -0.818

a PB 260 -0.8243

a PB 217 -1.135

**Variable: NINJA_6328**

Df Sum Sq Mean Sq F value Pr(>F)

clone 2 10.42 5.211 3.875 0.044 *

Residuals 15 20.17 1.345

---

Signif. codes: 0 ‘***’ 0.001 ‘**’ 0.01 ‘*’ 0.05 ‘.’ 0.1 ‘ ’ 1

Groups, Treatments and means

a RRIM 600 -2.238

ab PB 217 -2.604

b PB 260 -4.004

**Variable: TPL_7591**

Df Sum Sq Mean Sq F value Pr(>F)

clone 2 0.2632 0.13160 2.569 0.11

Residuals 15 0.7685 0.05123

Groups, Treatments and means

a PB 260 -3.241

a PB 217 -3.38

a RRIM 600 -3.537

**Variable: JAZ_14313**

Df Sum Sq Mean Sq F value Pr(>F)

clone 2 0.2505 0.12523 6.112 0.0114 *

Residuals 15 0.3073 0.02049

---

Signif. codes: 0 ‘***’ 0.001 ‘**’ 0.01 ‘*’ 0.05 ‘.’ 0.1 ‘ ’ 1

Groups, Treatments and means

a PB 260 -0.4251

b PB 217 -0.6573

b RRIM 600 -0.69

**Variable: JAR_14894**

Df Sum Sq Mean Sq F value Pr(>F)

clone 2 0.188 0.0940 0.167 0.848

Residuals 15 8.459 0.5639

Groups, Treatments and means

a PB 260 -2.613

a PB 217 -2.752

a RRIM 600 -2.863

**Variable: MED25_16787**

Df Sum Sq Mean Sq F value Pr(>F)

clone 2 0.9863 0.4931 24.15 2.04e-05 ***

Residuals 15 0.3063 0.0204

---

Signif. codes: 0 ‘***’ 0.001 ‘**’ 0.01 ‘*’ 0.05 ‘.’ 0.1 ‘ ’ 1

Groups, Treatments and means

a RRIM 600 -2.103

b PB 260 -2.366

c PB 217 -2.676

**Variable: JAZ_17062**

Df Sum Sq Mean Sq F value Pr(>F)

clone 2 1.5444 0.7722 23.97 2.13e-05 ***

Residuals 15 0.4833 0.0322

---

Signif. codes: 0 ‘***’ 0.001 ‘**’ 0.01 ‘*’ 0.05 ‘.’ 0.1 ‘ ’ 1

Groups, Treatments and means

a PB 260 -0.7258

b PB 217 -1.2

b RRIM 600 -1.429

**Variable: JAZ_19967**

Df Sum Sq Mean Sq F value Pr(>F)

clone 2 8.43 4.215 22.58 2.99e-05 ***

Residuals 15 2.80 0.187

---

Signif. codes: 0 ‘***’ 0.001 ‘**’ 0.01 ‘*’ 0.05 ‘.’ 0.1 ‘ ’ 1

Groups, Treatments and means

a PB 260 -0.6587

b PB 217 -1.402

c RRIM 600 -2.332

**Variable: JAR_20347**

Df Sum Sq Mean Sq F value Pr(>F)

clone 2 0.0404 0.02018 0.29 0.752

Residuals 15 1.0420 0.06947

Groups, Treatments and means

a PB 217 -2.978

a PB 260 -3.068

a RRIM 600 -3.086

**Variable: JAR_21367**

Df Sum Sq Mean Sq F value Pr(>F)

clone 2 6.562 3.281 1.588 0.237

Residuals 15 30.993 2.066

Groups, Treatments and means

a RRIM 600 -6.456

a PB 260 -7.629

a PB 217 -7.822

**Variable: JAZ_26925**

Df Sum Sq Mean Sq F value Pr(>F)

clone 2 16.43 8.214 1.769 0.204

Residuals 15 69.64 4.643

Groups, Treatments and means

a PB 260 -4.255

a RRIM 600 -4.922

a PB 217 -6.531

**Variable: JAZ_29511**

Df Sum Sq Mean Sq F value Pr(>F)

clone 2 0.0488 0.02441 0.34 0.717

Residuals 15 1.0760 0.07173

Groups, Treatments and means

a RRIM 600 -1.696

a PB 260 -1.759

a PB 217 -1.824

**Variable: JAR_59958**

Df Sum Sq Mean Sq F value Pr(>F)

clone 2 5.620 2.8102 30.47 5.22e-06 ***

Residuals 15 1.384 0.0922

---

Signif. codes: 0 ‘***’ 0.001 ‘**’ 0.01 ‘*’ 0.05 ‘.’ 0.1 ‘ ’ 1

Groups, Treatments and means

a RRIM 600 -1.591

b PB 217 -2.195

c PB 260 -2.957

**Variable: MYC_94937**

Df Sum Sq Mean Sq F value Pr(>F)

clone 2 30.01 15.005 4.833 0.024 *

Residuals 15 46.56 3.104

---

Signif. codes: 0 ‘***’ 0.001 ‘**’ 0.01 ‘*’ 0.05 ‘.’ 0.1 ‘ ’ 1

Groups, Treatments and means

a PB 217 -2.398

ab RRIM 600 -2.586

b PB 260 -5.226
