# Supplementary material for: Transcriptional and post-transcriptional regulation of the jasmonate signalling pathway in response to abiotic and harvesting stress in Hevea brasiliensis
Source: BMC Plant Biol. 2014 Dec 2;14:341. doi: 10.1186/s12870-014-0341-0 (PMC4274682; doi:10.1186/s12870-014-0341-0)
Supplement: Additional file 15: — Analysis of variance (ANOVA) to test effect of tapping, ethephon treatment, tree age and cross effect on the ratio spliced/unspliced of JAZ_1229 and JAZ-1660. [file 12870_2014_341_MOESM15_ESM.docx]

**Additional file 13**: Analysis of variance (ANOVA) to test effect of tapping, ethephon treatment, tree age and cross effect on the ratio spliced/unspliced of JAZ_1229 and JAZ-1660

| **Gene** | **Variable** | **df** | **Sum Sq** | **Mean Sq** | **F value** | **P (>F)** |
| --- | --- | --- | --- | --- | --- | --- |
| **JAZ_1229** | Tapping  Stimulation  Age  Tapping x Stimulation  Stimulation x Age  Residual | 2  1  1  1  1  4 | 0.3569  0.6476  0.1953  0.4900  0.0246  0.0645 | 0.1784  0.6476  0.1953  0.2450  0.0246  0.0161 | 11.059  40.137  12.103  15.184  1.526 | 0.02345  0.00318  0.02538  0.01355  0.28431 |
| **JAZ_1660** | Tapping  Stimulation  Age  Tapping x Stimulation  Stimulation x Age  Residual | 2  1  1  1  1  4 | 0.2705  0.5133  0.1793  0.0443  0.0050  0.0445 | 0.1352  0.5133  0.1793  0.0222  0.0050  0.0111 | 12.147  46.105  16.106  1.992  0.452 | 0.01999  0.00246  0.01595  0.25106  0.53814 |
